# Prolonged Isolated Soluble Dietary Fibre Supplementation in Overweight and Obese Patients: A Systematic Review with Meta-Analysis of Randomised Controlled Trials

**DOI:** 10.3390/nu14132627

**Published:** 2022-06-24

**Authors:** Valentina V. Huwiler, Katja A. Schönenberger, Alexander Segesser von Brunegg, Emilie Reber, Stefan Mühlebach, Zeno Stanga, Maria L. Balmer

**Affiliations:** 1 Department of Diabetes, Endocrinology, Nutritional Medicine and Metabolism, Inselspital, Bern University Hospital, University of Bern, 3010 Bern, Switzerland; katja.schoenenberger@extern.insel.ch (K.A.S.); alexander.segesservonbrunegg@students.unibe.ch (A.S.v.B.); emilie.reber@insel.ch (E.R.); zeno.stanga@insel.ch (Z.S.); 2 Division of Clinical Pharmacy and Epidemiology, Department of Pharmaceutical Sciences, University of Basel, 4031 Basel, Switzerland; stefan.muehlebach@unibas.ch; 3 Diabetes Center Bern (DCB), 3010 Bern, Switzerland; 4 Department of Biomedical Research, University Clinic of Diabetes, Endocrinology, Nutritional Medicine and Metabolism, Inselspital, Bern University Hospital, University of Bern, 3010 Bern, Switzerland

**Keywords:** body weight, dietary fibre, supplementation, microbiome, prebiotics, overweight, obesity, metabolism, metabolic disease, systematic review and meta-analysis

## Abstract

The prevalence of overweight and obesity is rising rapidly, currently affecting 1.9 billion adults worldwide. Prebiotic dietary fibre supplementation is a promising approach to improve weight loss and reduce metabolic complications in overweight and obese subjects due to modifications of the microbiota composition and function. Previous systematic reviews and meta-analyses addressing similar questions revealed discordant evidence and/or are outdated. We searched MEDLINE, Embase, Google Scholar, and forward and backward citations for randomised controlled trials (RCTs) with isolated soluble dietary fibre supplementation for at least 12 weeks in overweight and obese patients measuring body weight, published through April 2022. We expressed the results as mean differences (MDs) using the random-effects model of the metafor package in R and assessed risk of bias using the Cochrane RoB2 tool. We conducted the study according to the PRISMA guidelines and registered the protocol on PROSPERO (CRD42022295246). The participants with dietary fibre supplementation showed a significantly higher reduction in body weight (MD −1.25 kg, 95% CI −2.24, −0.25; 27 RCTs; 1428 participants) accompanied by a significant decrease in BMI, waist circumference, fasting blood insulin, and HOMA-IR compared to the control group. Certainty of evidence was high, paving the way for the implementation of isolated soluble dietary fibre supplementation into clinical practice.

## 1. Introduction

The prevalence of overweight and obesity has tripled worldwide between 1975 and 2016, reaching pandemic dimensions of 1.9 billion overweight adults (BMI ≥ 25 kg/m^2^) including 650 million obese (BMI ≥ 30 kg/m^2^) individuals [1]. Excess body weight raises the risk of non-communicable diseases such as type 2 diabetes mellitus, cardiovascular diseases, and several cancer types [2]. Overweight and its associated comorbidities impose a huge socioeconomic burden, amounting to USD 1.7 trillion in the United States of America alone due to increased morbidity and mortality as well as loss in productivity [3]. The rudimental cause of overweight is an imbalance between intake and expenditure of energy over an extended period of time. Consequently, first-line treatments include lifestyle modifications such as diet change and increase in physical activity. Nutritional interventions, namely very-low-calorie diets (VLCDs, around 450–800 kcal energy per day) [4] or intermittent fasting (food abstinence of typically ≥12 h) [5] have gained popularity in recent years; however, long-term success is often limited. Where lifestyle modifications fail, more invasive treatments including medication (e.g., lipase inhibitors, GLP-1 analogues) or bariatric surgery are initiated with highly variable weight loss rates [6]. Adjunct or alternative treatment concepts are needed to increase weight reduction, assure long-term success, and include non-responders of standard therapy.

Previous studies found inverse associations between whole-grain consumption and obesity [7] and interrelated comorbidities including type 2 diabetes [8] and cardiovascular diseases [9]. However, we need interventional studies in order to conclude whether these associations are due to a direct beneficial effect of whole-grains or a correlation between whole grain consumption and a healthy lifestyle in general. Whole grains are rich in important nutrients such as polyphenols, minerals, and dietary fibres (DFs). Whole grains are a substrate defined as “resistant to digestion (hydrolysis) by enzymes of man” [10]. DFs are health-associated, and the recommended intake for adults ranges from 20 g to 38 g per day in most countries worldwide [11,12,13,14,15,16,17]. However, surveys implied that the recommended levels are rarely met [18]. While insoluble DFs primarily increase the bulk volume, soluble DFs are dissolved in water and gastrointestinal fluids and are readily metabolised by the human gut microbiome. Specific recommendations on soluble and insoluble DF intake remain scarce. The gut microbiota and bacteria-derived metabolites (e.g., short-chain fatty acids, SCFAs) were described to play an important role in obesity and host metabolism regulation [19,20,21]. The loss in microbial diversity observed in obesity was linked to metabolic alterations including changes in SCFA abundance that may be improved with oral DF supplementation [22,23,24].

Previous clinical trials indicated a wide range of physiological improvements after dietary supplementation with soluble DFs, e.g., glycaemic control, lipid profile, blood pressure, and weight loss [25,26,27,28,29]. Stimulating the gut microbiome with soluble DFs, a potent prebiotic, could be a powerful way to improve metabolic parameters and increase weight reduction in the overweight and obese population. Food items with a high content of soluble DFs are black beans with 4.2 g per 100 g [30], avocados with 4.2 g per piece [31], and apples with 1.3 g per fruit [32]. Despite the ample evidence, health claims from the Food and Drug Administration (FDA, United States of America), European Food Safety Authority (EFSA), and the Food Standards Australia New Zealand (FSANZ) [19] for soluble DFs in general or for distinct types, e.g., inulin and beta-glucan, predominantly address risk reduction of cardiovascular disease. Supplementation with soluble DFs and their impact on obesity management in clinical practice remain controversial, and robust scientific evidence is lacking.

Various published systematic reviews and meta-analyses examining the effect of soluble DFs on body weight revealed promising results. Jovanovski et al. (*n* = 3877 participants) showed that viscous DF supplementation decreased body weight by 0.46 kg after a median treatment duration of 10 weeks independent of an energy-restricted diet in overweight and obese subjects with negligible effects in healthy subjects or people with elevated cardiovascular risk [29]. However, the inclusion of whole-food sources containing other health-related compounds makes it difficult to draw conclusions on the effect of soluble DFs themselves. The National Institute for Health and Care Excellence (NICE, UK) recommends a minimal duration of 12 weeks for weight management programmes [33]. Yet, about two thirds of the included studies were below this threshold. Furthermore, numerous studies with a short-term duration (<12 weeks) of DF supplementation failed to elicit major compositional changes in the gut microbiome [34], the primary target of soluble DF interventions. Studies with short-term intervention bear the risk of showing effects solely due to coincidence and not due to the intervention (causality). The meta-analysis by Thompson et al. (*n* = 609 participants) indicated an even greater effect with a mean weight reduction of 2.52 kg after supplementation with soluble DFs for 2 to 17 weeks [29]. Again, the value of the analysis was limited by the inclusion of studies with short-term intervention. Both meta-analyses investigated the effect of soluble DF supplementation in the absence of other interventions. In clinical practice, nutritional supplementation is rarely a stand-alone treatment for overweight and obesity, and therefore, elucidating the potential as an adjunct therapy is of major interest.

There is substantial need for a new systematic review and meta-analysis due to the abovementioned limitations and the high number of newly published clinical trials not covered in the previous meta-analyses. To our knowledge, this is the first systematic review and meta-analysis investigating the effect of prolonged (≥12 weeks) soluble DF supplementation on body weight and other metabolic parameters in overweight and obese people, non-restrictive in terms of the DF type and background treatment. We aimed to provide solid scientific recommendations on soluble DF supplementation in overweight and obese people in the clinical weight management. We hypothesised that soluble DF supplementation: (a) elicits a significant weight reduction and (b) improves obesity-related parameters; (c) multimodal therapy is more effective than single-component treatments; and outcomes are influenced by (d) study duration, (e) DF type, and (f) DF dose.

## 2. Materials and Methods

We conducted the present systematic review and meta-analysis according to the current Preferred Reporting Items for Systematic Reviews and Meta-Analyses (PRISMA) 2020 guidelines [35]. Procedures were conformed with the Cochrane Handbook for Systematic Reviews of Intervention [36] and the 4th Edition of the Research Synthesis and Meta-analysis book by Cooper [37]. Studies emphasising the effect of isolated soluble DF supplementation on body weight in overweight and obese adults were eligible. The protocol was published, and progress was documented on PROSPERO (CRD42022295246).

### 2.1. Search Strategy

We systematically searched the electronic databases MEDLINE via OVID, Embase via Elsevier, and Google Scholar from inception through April 2022. The search strings included MeSH terms, Emtree terms, and text words in title, abstract, and keywords containing the terms DF, overweight/obesity, and body weight. Appendix A lists the full search string for each database. Citations (forward citation search) and references (backward citation search) of all included studies were identified using Web of Science through April 2022. Reports included in two previous systematic reviews and meta-analyses [29,30] addressing a similar research question were assessed for eligibility. We deduplicated records using EndNote’s (version 20) automation feature.

### 2.2. Study Eligibility

We included: (a) randomised, controlled clinical trials (RCT), (b) published in (c) English, German, or French with (d) no restriction on publication date. We limited the study population to (e) adults (≥18 years) with (f) overweight or obesity (BMI ≥ 25 kg/m^2^ for Caucasians and ≥23 kg/m^2^ for Asians). Studies with a (g) DF supplementation of at least 2 g and a (h) duration of at least 12 weeks that (i) measured baseline and final body weight were included. The studies were non-eligible if: (a) dietary supplements contained less than 50% soluble DFs or in case the (b) intervention and control compound showed major differences in macro- and/or micronutrient composition in addition to that of -DFs. Previous (c) weight loss phases without DFs that stopped during the DF supplementation period disqualified the studies. We excluded (d) observational studies, conference abstracts and proceedings, case reports, editorials, letters, and notes. The two authors V.H. and A.S. independently screened titles and/or abstracts for eligibility. We retrieved full-text articles of the potentially eligible studies, and the two authors separately assessed compliance with inclusion and exclusion criteria. Interrater disagreements were resolved through a consensus or discussion with a third author.

### 2.3. Data Extraction and Risk of Bias Assessment

We extracted the following data with a standardised, pilot-tested coding sheet:

- Report: title, author, publication journal, Digital Object Identifier (DOI);
- Study: design, intention-to-treat or per-protocol analysis;
- Participants: group size, BMI range, age, sex distribution;
- Intervention: DF, placebo, dose, intervention duration, mode of delivery, background treatment;
- Outcome: mean and standard deviation of the outcomes at baseline and end of the intervention.

The primary outcome was body weight [kg], and secondary outcomes were BMI [kg/m^2^], body fat [%], waist circumference [cm], HbA1c [%], HOMA-IR [38], fasting blood glucose [mmol/L], fasting blood insulin [mIU/L], C-reactive protein (CRP) [mg/L], and stool calprotectin [μg/g faeces]. Multiple cohorts from the same report were coded individually. If several records on the same cohort were published containing different outcome data, we combined them into a single study. We selected the results of the longest intervention period if multiple time points were presented in a record. Two authors (V.H. and A.S.) extracted the data independently, and missing data were requested from the authors directly. Interraters’ accordance on continuous variables was above 0.95. Two authors independently assessed the risk of bias for the assignment to intervention (‘intention-to-treat’ effect) was assessed in terms of randomisation process, deviation from intended intervention, missing outcome data, outcome measurement, and selection of reported results for each study using the revised Cochrane risk of bias tool for randomised trials (RoB 2) [39].

### 2.4. Data Synthesis and Analysis

If more than four studies reported a specific outcome, we conducted a meta-analysis using the *metafor* package [40] in R software version 4.1.2 (R Core Team (2022). R: A language and environment for statistical computing. R Foundation for Statistical Computing, Vienna, Austria. Available from: https://www.R-project.org, accessed on 15 May 2022) [41]. All units were converted to the predefined units before analysis. Missing standard deviations of the final measurement of the primary outcome (body weight) were imputed from the baseline and change of standard deviations [36]. We statistically synthesised the data as mean differences (MD) using the random-effects model with the Hedges estimator [42,43] accounting for systematic variation of effect sizes across studies. We combined CRP and high-sensitivity CRP (hs-CRP) as we used MD and correlation among the two parameters is expected to be near 1 [32]. Additionally, we calculated the pooled CRP standardised MD to verify the MD results. We weighted the studies using the inverse-variance method [36] adjusted for the study size to prevent underestimation of studies with a high variance caused by the background treatment (e.g., bariatric surgery). We calculated the Q statistics according to Cochrane [44] and the I^2^ statistic [45] to estimate heterogeneity between the studies. We conducted a moderator analysis for intervention duration, dose, and DF type with a meta-regression model for the primary outcome of body weight. The publication bias was assessed based on funnel plot asymmetry by carrying out the Egger’s regression test [46] and Rank correlation test [47]. We carried out outlier and influential case diagnostics by visually inspecting the Baujat plot [48] and calculating the leave-one-out diagnostic for the externally standardised residual, DFFITS value, Cook’s distance, covariance ratio, amount of (residual) heterogeneity, test statistics of the test for (residual) heterogeneity, and DFBETAS value using the influence function of the metafor package. To conclude, we assessed certainty of evidence for the primary outcome, body weight, with the grading of recommendation assessments, development, and evaluation (GRADE) [49] approach using the GRADEpro GDT software (GRADEpro GDT (2022). GRADEpro Guideline Development Tool [Software]. McMaster University and Evidence Prime, 2022. Available from: https://www.gradepro.org, accessed on 15 May 2022) [50].

## 3. Results

### 3.1. Study Selection

**Figure 1:**
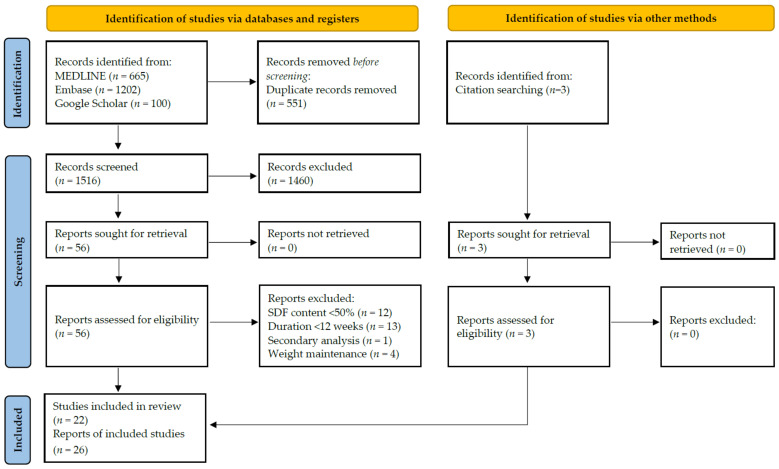
PRISMA flow chart according to [1] elaborating on the selection process of the 22 included studies. SDF = soluble dietary fibre.

Figure 1 summarises the selection process of eligible studies. We detected 1516 unique records whereof we screened title and/or abstract, assessed 56 full-text reports, and finally included 26 reports (22 studies). We excluded 30 studies from our review either because soluble DF content of supplement was below 50% (*n* = 12), intervention duration was below 12 weeks (*n* = 13), the subgroup of an included study was analysed (secondary analysis; *n* = 1), or weight loss phase without DF supplementation that stopped during intervention preceded the study (*n* = 4). Three additional reports were included after searching the 1130 records that cited the included studies and the 823 references of the included studies using Web of Science. The study by Cicero et al. [51] was not available on Web of Science and we used Google Scholar for citation searching. In total, we included 26 reports covering 22 studies and 27 cohorts. Of the 22 included studies, only 5 and 3 were covered in the previous meta-analyses by Jovanovski et al. [30] or Thompson et al. [29], respectively.

### 3.2. Study and Participant Characteristics

All 22 included studies were parallel RCTs published between 2006 and 2022. Overall, 90% of the studies were double-blinded (*n* = 20). Table 1 lists the study and participant characteristics of the included trials. Intervention duration ranged from 12 to 52 weeks (mean = 17 weeks, median = 12 weeks) with supplemented DF doses ranging from 2.6 g to 29 g per day (mean = 11 g/day). Eleven cohorts received soluble DF supplementation in the absence of other interventions, whereas 16 cohorts received an adjunct treatment, i.e., energy-restricted diet (*n* = 13), bariatric surgery (*n* = 1), and/or increased physical activity (*n* = 1).

**Table 1:** Study and participant characteristics of the 22 included randomised controlled trials.

Author, Year	Intervention, Dose	Duration [Weeks]	Study Population BMI [kg/m^2^] Age [Years]	Comorbidities	Sample Size (Intervention/Control); % Female	Outcome Measures Evaluated									Additional Treatment	Country
						BW	BM	BF	WC	Hb	HO	BG	BI	CR		
Bomhof, 2019 [52]	FOS orally, 8–16 g/day	36	BMI ≥ 25 (Caucasian) or 23 (Asians) Age ≥ 18	NASH	*n* = 14 (8/6); 42.8% female	x	x		x		x	x	x		U	Canada
Bongartz, 2022 [53]	Flaxseed mucilage powder in water, 5.1 g/day	12	BMI 25–35 Age 18–65	NS	*n* = 67 (34/33); 70.1% female	x	x		x		x	x	x		ERD	Germany
	Flaxseed mucilage powder in water, 2.6 g/day	12	BMI 25–35 Age 18–65	NS	*n* = 66 (33/33); 65.2% female	x	x		x		x	x	x		ERD	Germany
Calikoglu, 2021 [54]	Inulin + FOS powder in yogurt, 10 g/day	26	BMI ≥ 35 Age NR	NS	*n* = 30 (14/16); NR females	x	x			x	x	x	x		BS	Turkey
Cicero, 2010 [51]	Guar gum orally, 7 g/day	26	BMI 25–30 Age 50–70	Metabolic syndrome	*n* = 91 (46/45); 52.8% female	x			x	x	x	x	x		ERD	Italy
Dewulf, 2013 [55]	Inulin + FOS powder in fluid, 16 g/day	12	BMI ≥ 30 Age 18–65	NS	*n* = 30 (15/15); 100% female	x	x	x	x	x	x	x	x		U	Belgium
Genta, 2009 [56]	FOS syrup, 0.14 g/kg BW/day	17	BMI ≥ 30 Age 31–49	Dyslipidemia, constipation	*n* = 35 (20/15); 100% female	x	x		x		x	x	x		ERD, no FOS rich food	Argentina
Grube, 2013 [57]	Litramine IQP G-002AS tablets, 3 g/day	12	BMI 25–35 Age 18–60	NS	*n* = 118 (59/59); 75.6% females	x	x	x	x						ERD, PA	Germany
Grunberger, 2007 [58]	α-Cyclodextrin tablets, 6 g/day	12	BMI ≥ 30 Age ≥ 30	T2D	*n* = 47 (20/27); 53.2% female	x	x			x				x	U	USA
Guérin-Deremaux, 2011 [59,60]	Nutriose ^2^ powder in fruit juice, 29 g/day	12	BMI 24–28 Age 20–35	NS	*n* = 113 (57/56); 0% female	x	x								U	China
Hassan, 2020 [61]	Inulin + RS pasta, 15 g/100 g pasta/day	12	BMI ≥30 Age 43.4 ± 10.3 ^1^	NS	*n* = 30 (15/15); 80.0% female	x	x			x		x	x		ERD	Italy
Hess, 2020 [62,63]	Inulin powder in milk, 20 g/day	12	BMI 28–45 Age 18–60	NS	*n* = 86 (42/44); 64.0% female	x	x	x	x	x	x	x	x	x	ERD, FI	Denmark
Jensen, 2012 [64]	Alginate powder in water, 15 g/day	12	BMI 30–45 Age 20–55	NS	*n* = 80 (38/42); 67.5% female	x	x	x	x	x	x	x	x	x	ERD, Calcium	Denmark
Kristensen, 2017 [65]	Flaxseed DF powder in fluid or food, 5 g/day	12	BMI 30–40 Age 20–60	NS	*n* = 16 (10/6); 80.7% female	x			x	x	x	x	x	x	ERD, Orlistat, Vitamins	Denmark
	Flaxseed DF powder in fluid or food, 5 g/day	12	BMI 30–40 Age 20–60	NS	*n* = 22 (10/12); 80.6% female	x			x	x	x	x	x	x	ERD, Orlistat, Vitamins, Calcium	Denmark
Pal, 2016 [66]	PGX ^3^ powder in water, 15 g/day	52	BMI 19–68 Age 25–47	NS	*n* = 57 (25/32); 53.6% female	x ^4^									U	Australia
Parnell, 2009 [67,68]	FOS powder in fluid, 21 g/day	12	BMI > 25 Age 20–70	NS	*n* = 39 (21/18); 82.0% female	x		x	x						U	Canada
Pol, 2018 [69]	FOS bar, 16 g/day	12	BMI 25–35 Age 20–60	NS	*n* = 55 (29/26) 65.5% female	x		x	x						U	Netherlands
Reimer, 2021 [70]	PGX ^3^ sprinkled on food, 15–20 g/day	52	BMI 27–60 Age 18–75	T2D	*n* = 100 (53/47); 68.3% female	x	x		x	x					ERD	Canada
Reimer, 2017 [71]	FOS + Inulin bar, 8 g/day	12	BMI > 25 Age 18–75	NS	*n* = 53 (26/27); 54.2% female	x	x	x	x	x					U	Canada
	FOS + Inulin protein bar, 8 g/day	12	BMI > 25 Age 18–75	NS	*n* = 43 (21/22); 51.5% female	x	x	x	x	x					U	Canada
Reimer, 2013 [72]	PGX ^3^ powder with yogurt, 15 g/day	14	BMI 24–30 Age 20–65	NS	*n* = 64 (32/32); 56.3% female	x	x		x		x	x	x		U	Japan
Solah, 2017 [73]	PGX ^3^ softgel capsules with water, 8–11 g/day	12	BMI 25–35 Age 25–70	NS	*n* = 51 (32/19); 77.5% female	x ^4^									U	Australia
	PGX ^3^ granules in food or fluid, 12 g/day	12	BMI 25–35 Age 25–70	NS	*n* = 51 (32/19); 76.3% female	x ^4^									U	Australia
Tovar, 2012 [74]	Inulin powder in milk with PMR powder, 10 g/day	12	BMI > 25 Age 18–50	NS	*n* = 51 (23/28); 100% female	x									ERD, PMR	Mexico
	Inulin powder in fluid, 10 g/day	12	BMI > 25 Age 18–50	NS	*n* = 59 (30/29); 100% female	x									ERD	Mexico
Wood, 2006 [75,76]	Glucomannan capsules with water, 3 g/day	12	BMI 25–35 Age 20–69	NS	*n* = 29 (14/15); 0% female	x	x	x	x		x	x	x	x	ERD, vitamins	USA

Age = age range of included subjects or ^1^ mean ± SD; ^2^ resistant corn dextrin, ^3^ combination of glucomannan, sodium alginate, and xanthan gum; ^4^ SD after 12 weeks estimated according to Cochrane; BG = fasting blood glucose; BI = fasting blood insulin; BM = BMI; BS = bariatric surgery; BW = body weight; CR = C-reactive protein; DF = dietary fibre; ERD = energy-restricted diet; FI = recommendation on dietary fibre intake; FOS = fructooligosaccharides; Hb = HbA1c; HO = HOMA-IR; NASH = non-alcoholic steatohepatitis; NR = not reported; NS = not specified; NT = no treatment; PA = physical activity; PMR = partial meal replacement; RS = resistant starch; T2D = type 2 diabetes; U = usual diet and physical activity; WC = waist circumference.

Table S1 lists the control treatments and raw data on body weight. Of the 22 studies, 11 (50%) were funded by industry, 9 (41%) by universities or governments, and 2 (9%) by industry and government combined (Table S1). We included a total of 1428 participants in this review covering an age range from 18 to 75 years and BMI from 25 to 68 kg/m^2^. On average, the cohorts included 66% women, with 4 cohorts (15%) containing exclusively female [55,56,74] participants and 2 cohorts (7%) consisting of only male [59,76] participants. One cohort (4%) did not report the sex distribution of participants [53] (Table 1). We processed intention-to-treat results except for the study by Reimer et al. [70], which only reported the per-protocol results. We retrieved missing results from all authors directly except for the studies by Solah et al. [73] and Pal et al. [66]. The missing data for Solah et al. were imputed according to Cochrane, and MD and CI reported in the study by Pal et al. were added directly to the meta-analysis. The two cohorts from the studies by Solah et al. [73] and Bongartz et al. [52] shared a control group that we only counted once for the sum of pooled participants for each outcome.

### 3.3. Quality and Risk of Bias of Primary Outcome Body Weight

**Figure 2:**
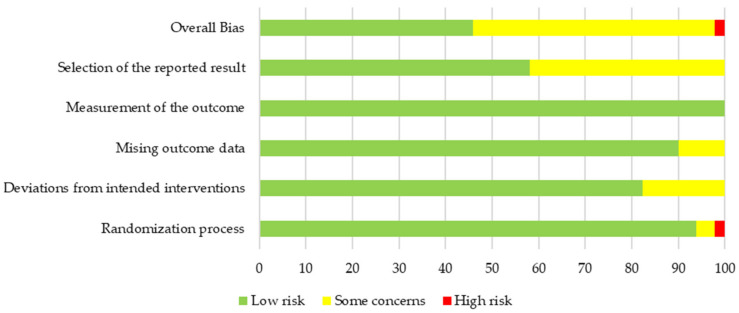
Overall risk of bias results for the primary outcome of body weight.

**Figure 3:**
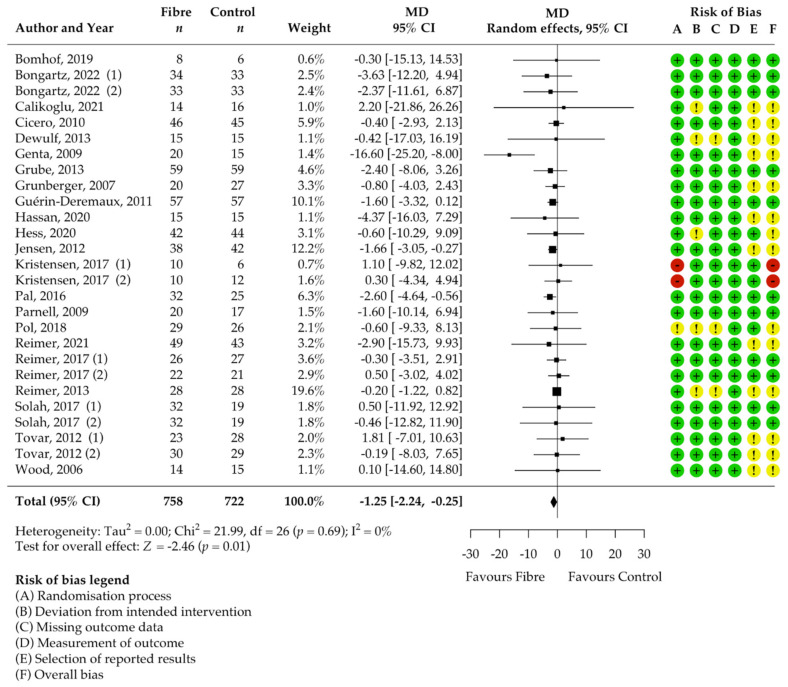
Isolated soluble dietary fibre supplementation significantly decreased body weight [kg] in overweight and obese people (*n* = 1428). Black rectangles represent MD for each study; the size of the rectangle is proportional to the weight of the study for the pooled effect. Horizontal lines indicate 95% CI. The black diamond summarises the pooled MD data. MD = mean difference; (1)/(2) indicate cohort 1 and 2 of study [51,52,53,54,55,56,57,58,59,60,61,62,63,64,65,66,67,68,69,70,71,72,73,74,75,76].

Overall risk of bias was low for 11 cohorts (41%), high for 2 cohorts (7%) and there were some concerns for 14 cohorts (52%). The majority of concerns (42%) arose from the selection of the reported results because no protocol was published on a non-commercial trial registry before study start (Figure 2 and Figure 3). The two cohorts indicating a high risk of bias belonged to the same study [65]. The allocation was not concealed but performed by a researcher of the team during the randomisation visit. As the randomisation list was created before commencement of the study and both staff and participants were blinded during the study, consequences of the open allocation were expected to be reasonable. Additionally, excluding the two cohorts led to similar pooled results (MD −1.29 kg, 95% CI −2.30, −0.28). No significant reporting bias was identified by asymmetry analysis of the funnel plot using the Egger’s and the Rank’s test (*p* = 0.87 and *p* = 1.00, respectively, Table 2). Additionally, we visually did not detect any asymmetry of the funnel plot (Figure S8).

### 3.4. Pooled Results on Primary Outcome Body Weight

The results of the meta-analysis showed a significant decrease in body weight after supplementation with soluble DFs for at least 12 weeks compared to the control group (MD −1.25 kg, 95% CI −2.24, −0.25; *p* = 0.01; I^2^ = 0%; 27 RCTs; 1428 participants, Table 2, Figure 3). We confirmed statistical significance by pooling the standardised MD, known as Cohen’s d [77] (standardised MD −0.20, 95% CI −0.34, −0.05; *p* = 0.01).

There was no significant heterogeneity among the studies (I^2^ 0%, 95% CI 0.00, 52.46; τ^2^ 0, 95% CI 0.00, 3.39; χ^2^ 22.00; *p* = 0.69, Figure 3). Patients included in the individual studies showed similar characteristics and patients of 22 cohorts were considered healthy or had no specified comorbidities whereas 2 cohorts included only patients with type 2 diabetes, one cohort only metabolic syndrome patients, one only non-alcoholic steatohepatitis patients, and one only had patients with dyslipidaemia and constipation (Table 1). Genta et al. [56] showed with −16.60 kg the highest MD (95% CI −25.50, −8.00) for weight reduction over all eligible studies and was identified as an outlier in the Bajut plot (Supplementary S3). However, we confirmed significance of pooled effect (MD −1.02 kg, 95% CI −2.03, −0.02; *p* = 0.04) even when this study was excluded and influential case diagnostics did not classify the study as a significantly influential study. Therefore, we included this study in the final analysis. The dose of DF significantly impacted the effect (*p* = 0.03). Higher soluble DF doses tended to decrease body weight more than lower doses (Figure S7). There was no significant influence of the intervention duration (*p* = 0.20) and soluble DF type (*p* = 0.94) on the amount of body weight reduction. Participants with supplementation of DF alone benefited as much as participants with an adjunct weight management treatment (*p* = 0.17). Despite the lack of significance, subgroup analysis suggested that adjunct soluble DF supplementation may reduce body weight more than exclusive supplementation (MD −1.90 kg, 95% CI −3.65, −0.15 vs. MD −0.77, 95% CI −1.47, −0.06). The proportion of women in the study had no significant effect (*p* = 1.00).

We estimated the certainty of the evidence to be high due to the inclusion of only RCT and the low risk of bias, inconsistency, indirectness, and imprecision (“not serious”, Appendix B Table A1).

### 3.5. Pooled Results of Secondary Outcomes (BMI, Body Fat, Waist Circumference, HbA1c, HOMA-IR, Fasting Blood Glucose, Fasting Blood Insulin, CRP, and Stool Calprotectin)

**Figure 4:**
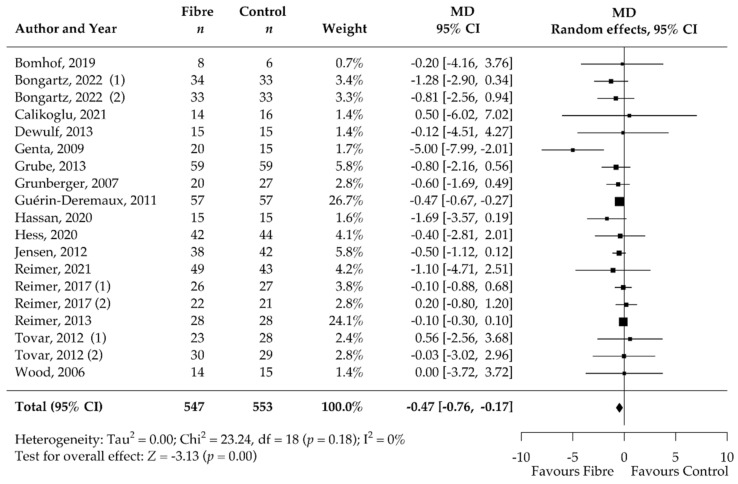
Isolated soluble dietary fibre supplementation significantly decreased BMI [kg/m^2^] in overweight and obese people (*n* = 1067). Black rectangles represent MD for each study; the size of the rectangle is proportional to the weight of the study for the pooled effect. Horizontal lines indicate 95% CI. The black diamond summarises the pooled MD data. MD = mean difference; (1)/(2) indicate cohort 1 and 2 of study [52,53,54,55,56,57,58,59,60,61,62,63,64,70,71,72,73,74,75,76].

**Figure 5:**
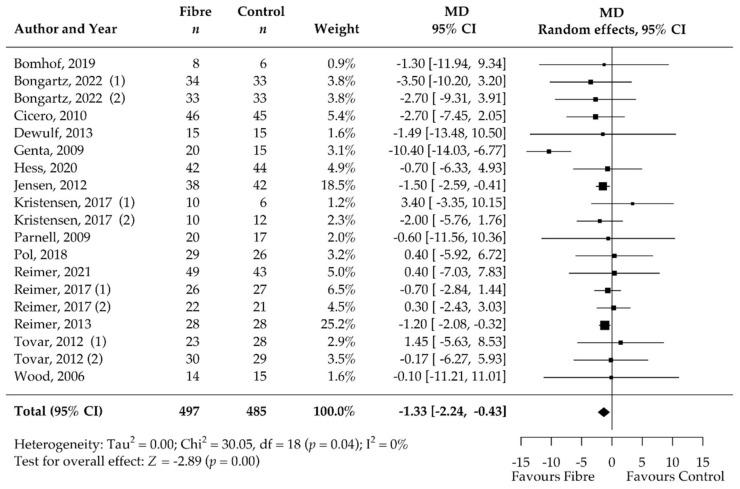
Isolated soluble dietary fibre supplementation significantly decreased waist circumference [cm] in overweight and obese people (*n* = 949). Black rectangles represent MD for each study; the size of the rectangle is proportional to the weight of the study for the pooled effect. Horizontal lines indicate 95% CI. The black diamond summarises the pooled MD data. MD = mean difference; (1)/(2) indicate cohort 1 and 2 of study [51,52,53,55,56,62,63,64,65,67,68,69,70,71,72,74,75,76].

**Figure 6:**
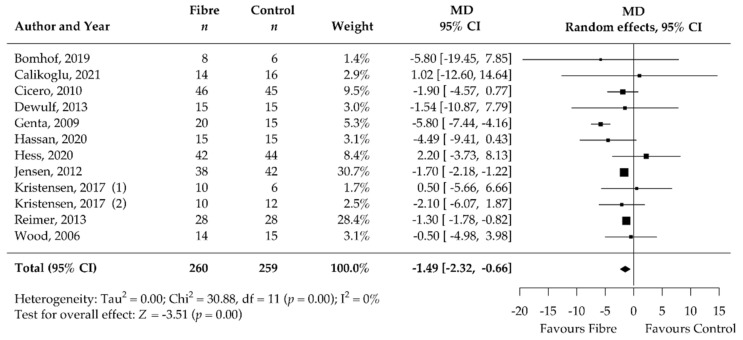
Isolated soluble dietary fibre supplementation significantly decreased fasting blood insulin [mIU/L] in overweight and obese people (*n* = 519). Black rectangles represent MD for each study; the size of the rectangle is proportional to the weight of the study for the pooled effect. Horizontal lines indicate 95% CI. The black diamond summarises the pooled MD data. MD = mean difference; (1)/(2) indicate cohort 1 and 2 of study [51,52,54,55,56,61,62,63,64,65,70,71,72,75,76].

**Figure 7:**
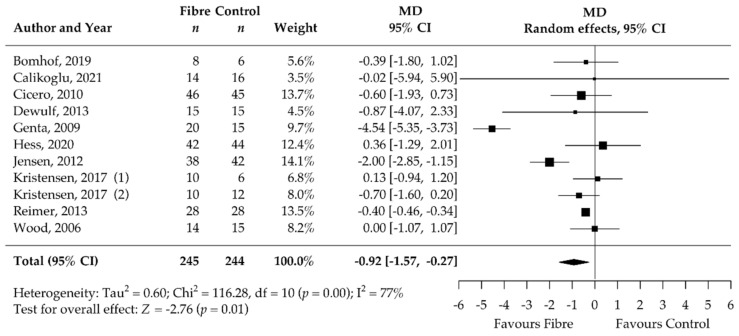
Isolated soluble dietary fibre supplementation significantly decreased HOMA-IR in overweight and obese people (*n* = 489). Black rectangles represent MD for each study; the size of the rectangle is proportional to the weight of the study for the pooled effect. Horizontal line indicates 95% CI. The black diamond summarises the pooled MD data. MD = mean difference; (1)/(2) indicate cohort 1 and 2 of study [51,52,54,56,62,63,64,65,70,71,72,75,76].

Analysis of the secondary outcomes revealed a significant decrease in BMI (MD −0.47 kg/m^2^, 95% CI −0.76, −0.17; *p* < 0.001; I^2^ = 0%; 19 RCTs; 1067 participants, Table 2, Figure 4) and waist circumference (MD −1.33 cm, 95% CI −2.24, −0.43; *p* < 0.001; I^2^ = 0%; 19 RCTs; 949 participants, Table 2, Figure 5) after soluble DF supplementation. Moreover, fasting blood insulin significantly decreased (MD −1.49 mIU/L, 95% CI −2.32, −0.66; *p* < 0.001; I^2^ = 0%; 12 RCTs; 519 participants, Table 2, Figure 6) as well as HOMA-IR (MD −0.92, 95% CI −1.57, −0.27; *p* = 0.01; I^2^ = 77%, 11 RCTs, 489 participants, Table 2, Figure 7). There was no significant effect on the percentage of body fat, fasting blood glucose, HbA1c, and CRP (Table 2, Figures S2–S6). None of the eligible studies examined stool calprotectin levels.

## 4. Discussion

This systematic review and meta-analysis showed that supplementation with isolated soluble DFs for at least 12 weeks significantly decreased body weight compared to the control without soluble DF supplementation (MD −1.25 kg, 95% CI −2.24, −0.25; *p* = 0.01; I^2^ = 0%; 27 RCTs; 1428 participants) in overweight and obese people. Our findings agree well with previous meta-analyses addressing similar research questions. In 2017, Thompson et al. observed an MD of −2.52 kg (95% CI −4.25, −0.79; *p* = 0.004, I^2^ = 96%; 10 RCTs; 520 participants) after supplementation with isolated soluble DFs in overweight and obese participants [29]. The higher MD resulted mainly from including only 10 studies. Thus, the outlier study by Genta et al. [56] had a stronger impact on the pooled results.

In 2020, Jovanovski et al. showed an MD of −0.46 kg (95% CI −0.75, −0.18; *p* < 0.00001, I^2^ = 88%; 18 RCTs; 742 participants) after viscous DF supplementation in overweight and obese participants [30]. Neither study exploited the potential of DF supplementation as an adjunct treatment, and both lacked detailed long-term investigation (≥12 weeks), which is crucial in obesity management. Since then, new clinical trials have been carried out leading to a higher number of participants and more robust evidence that we included in our meta-analysis.

Increased body weight (BMI > 25 kg/m^2^) causes a substantial disease burden causing huge economic costs. Even a modest weight reduction (5%) is considered clinically relevant and has shown to reduce risk of obesity-related comorbidities and improve metabolic parameters such as serum triglycerides, blood glucose, and HbA1c [78,79,80]. For every kilogram of body weight lost, the risk for type 2 diabetes decreased by 16% [81], and α-diversity of the gut microbiome increased together with a reduced intestinal permeability [82] in previous studies. The intactness of the intestinal barrier is strongly related to low-grade systemic inflammation which is often observed in obesity-related diseases [83]. Current guidelines target a weight reduction of 3% to 5%, corresponding to 2.7 kg to 4.5 kg in our study population (median weight = 89.4 kg) [5,80]. Conventional weight reduction strategies resulted in highly variable success rates ranging from 1.1 kg for exercise (3 to 12 months) [84], 4.6 kg for diets (12 months) [85], 2.6 to 8.8 kg for pharmacotherapies (12 months) [86], and 26 kg for bariatric surgery [87]. The weight reduction by −1.0 kg observed after soluble DF supplementation in our study is comparable to the effect achieved by exercise. Interestingly, when the supplementation is used in addition to treatments such as energy-restricted diet or bariatric surgery, an even higher reduction in body weight (1.9 kg) was observed. Additionally, there was a slightly but significantly greater weight loss with increased dose of DF supplementation.

We additionally observed a reduction in BMI (−0.47 kg/m^2^) and waist circumference (−1.33 cm). The meta-analysis of the secondary outcomes predominantly confirmed the expected metabolic improvements associated with a moderate weight reduction of 1.25 kg. Fasting blood insulin (−1.49 mIU/L) and HOMA-IR (−0.92) significantly improved after soluble DF supplementation compared to control treatment. Our meta-analysis showed no significant effect of DF supplementation on CRP (inflammation) levels, fasting blood glucose, and HbA1c. This could be due to the inclusion of overweight and obese participants who were mostly without comorbidities. Therefore, the participants exhibited only limited systemic inflammation and had CRP levels within the normal range at baseline (mean CRP at baseline = 3.9 mg/L). Additionally, the median duration of only 12 weeks may be insufficient to observe significant changes in HbA1c, due to its long responding time. It remains to be shown whether the metabolic improvements were modulated directly by the DFs or were a consequence of the decreased body weight.

The mechanism by which DFs cause weight reduction is still a matter of debate. On the one hand, there is evidence that DFs exert a direct effect on the composition and function of the gut microbiome. DFs serve as substrates for different microbial communities and can result in higher SCFA levels [88]. For example, supplementation with resistant starch enriches specific bacterial groups such as *Bifidobacterium adolescentis*, *R. bromii*, and *Eubacterium rectale*, which are known SCFA producers [89,90,91]. SCFAs can regulate satiety by enhancing the production of key anorectic hormones such as peptide tyrosine tyrosine (PYY) and glucagon-like peptide-1 (GLP-1) [92]. Additionally, SCFAs acetate and propionate were shown to stimulate the production of the hormone leptin via white adipose tissue [93,94]. These mechanistic studies were mostly limited to preclinical investigations. On the other hand, Roager et al. found no major changes in the faecal microbiome in participants after increased whole grain intake for 8 weeks (179 ± 50 g/day) compared to a diet high in refined grain, despite significant body weight reduction [95]. Suhr et al. suggested that weight reduction after increased DF consumption was rather caused by decreased energy intake [96]. However, human studies investigating the satiation effect of DFs with ad libitum test meals are scarce and remain inconclusive [97,98,99].

Of the 22 included studies in our systematic review, only five examined gut microbiota composition changes [51,55,62,70,71]. The five studies that supplemented inulin-type fructans (i.e., inulin, fructooligosaccharides) observed a significant increase in *Bifidobacterium* abundance [51,55,62,71], and the study administering a combination of glucomannan, sodium alginate, and xanthan gum elicited an increase in *Collinsella*, *Parabacteroides*, and *Roseburia* genera [70]. These findings suggest a DF type-specific modulation of the gut microbiome. However, further long-term interventional studies with soluble DFs are needed to verify these findings and to draw conclusions on the sustainability of the modulations. Additionally, more trials investigating metabolic parameters in combination with microbiome composition and function may clarify the causality.

A broad body of evidence suggested that soluble DFs may be potent pharmaconutrients to decrease body weight in overweight and obese people with a high benefit–cost ratio. It is non-invasive and implementation is straightforward, and soluble DFs are well tolerated by the common population. The supplementation showed high potential as a stand-alone treatment and could even evoke additional weight reduction as an adjunct therapy.

The results of this meta-analysis support promising effects at doses as low as 3 g per day [52,57] with increasing effects at higher doses. Food items such as whole grains, beans and legumes, nuts and seeds, vegetables, and fruits are valuable sources of soluble DF. Caution is advised for amounts higher than 25 g of soluble DFs per day due to digestive adverse effects [56]. These amounts, corresponding to 6 avocados or 20 apples per day, are rarely met by consumption of high soluble DF foods [19,32,33]. Nonetheless, gradual increase in the daily soluble DF dose could ease gastrointestinal discomfort such as flatulence, diarrhoea, severe abdominal distention, and nausea [51,55,70,71]. Increased intake for at least 12 weeks is recommended to obtain comparable effects in a wide range of the population as indicated by the low heterogeneity of the results in our meta-analysis.

One of the strengths of this meta-analysis is the high certainty and robustness of the evidence on weight reduction. The homogeneous results indicate applicability to a wide range of obese and overweight people. However, the study had certain limitations. We focused on the body weight reduction as a primary outcome. The degree of weight reduction is highly dependent on the initial body weight. Therefore, the BMI or reduction of BMI z-score may be more meaningful study variables. Moreover, it would be highly interesting to better understand the characteristics and obesity-related diseases of patients who benefit most from DF supplementation. Unfortunately, we could not address this question in sufficient detail as most included studies examined overweight and obese participants without comorbidities. Lastly, the optimal intervention duration remains unclear because most studies lasted for 12 weeks, which was the minimum duration allowed for inclusion. Some questions remain open, and further research should address the long-term success in weight reduction and examine if further weight gain can be prevented by DF supplementation. Additionally, microbiome analyses are needed to better understand the mechanistic relationship between DF consumption and composition and function of the gut microbiome.

## 5. Conclusions

Isolated soluble DF supplementation significantly decreased body weight in overweight and obese participants. Mean weight reduction amounted to 1.25 kg over an average time of 17 weeks, even in addition to other weight reduction therapies. Furthermore, the treatment resulted in metabolic improvements such as fasting blood insulin and HOMA-IR. The high certainty of evidence indicates that increasing soluble DF consumption is an appropriate component of standard obesity therapy.

## Figures and Tables

**Table 2:** Pooled mean difference and CI, heterogeneity I^2^, and publication bias of included cohorts.

Outcome	Included Cohorts [*n*]	Pooled MD [95% CI]	Heterogeneity I^2^ [%]	Publication Bias	
				Egger’s [*p*]	Rank’s [*p*]
Body weight [kg]	27	**−1.25 [−2.24, −0.25]**	0	0.87	1.00
BMI [kg/m^2^]	19	**−0.47 [−0.76, −0.17]**	0	NA	NA
Body fat [%]	13	−0.37 [−2.46, 1.71]	0	NA	NA
Waist circumference [cm]	19	**−1.33 [−2.24, −0.43]**	0	NA	NA
HbA1c [%]	12	−0.11 [−0.23, 0.01]	0	NA	NA
HOMA-IR	11	**−0.92 [−1.57, −0.27]**	77	NA	NA
Fasting blood glucose [mmol/L]	14	−0.08 [−0.17, 0.01]	0	NA	NA
Fasting blood insulin [mIU/L]	12	**−1.49 [−2.32, −0.66]**	0	NA	NA
CRP [mg/L]	7	0.02 [−0.64, 0.67]	0	NA	NA

Bold values highlight significant outcome. CRP = C-reactive protein; NA = not assessed; MD = mean difference.

## Data Availability

All data supporting the reported results can be found in this publication and the Supplementary Materials.

## Supplementary Materials

The following supporting information can be downloaded at: https://www.mdpi.com/article/10.3390/nu14132627/s1, Figure S1. Forest plot SMD body weight; Figure S2. Forest plot MD percentage of body fat; Figure S3. Forest plot MD HbA1c; Figure S4. Forest plot MD fasting blood glucose; Figure S5. Forest plot MD CRP; Figure S6. Relationship of supplementation dose and mean difference of body weight; Figure S7. Publication bias analysis with the funnel plot for the primary outcome of body weight; Figure S8. Bajut plot for the outcome body weight; Figure S9. Influential analysis according to the influence function of the metaphor package in R; Table S1. Excluded studies according to Figure 1; Table S2. Study and participant characteristics of the 22 included RCTs.

## Appendix A

Search Strings for MEDLINE(R) ALL via OVID covering results from 1946 to 13 April 2022.

1. exp adiposity/or exp Obesity/(249464)
2. (adipos* or obes* or (excess adj3 weight) or overweight).ti,ab,kw. (449982)
3. exp dietary fiber/or exp Inulin/or exp xylans/or exp beta-Glucans/or exp beta-Glucans/or exp Pectins/or exp guar gum/or exp 4’-galactooligosaccharide/or exp fructooligosaccharide/or exp Psyllium/or exp Psyllium/or exp Flax/(50110)
4. ((diet* adj4 fib*) or (Wheat adj bran*) or Roughage* or “alimentary fib*” or “opti?fib*” or “stimulance multi fib*” or alant* or “dahlin” or inulin* or “synanthrin” or xylan* or arabinoxylan or xyloarabinan or hemixylan or (beta and glucan*) or “beta dextroglucan” or macrogard or Pectin* or Methoxy?pectin or guar or Glucotard or “GU-052” or slocose or supercol or “Cal-Ban 3000” or “burtonite v 7 e” or “cyamopsis gum” or decorpa or fibraguar or galactasol or “gum cyamopsis” or “hepart hp 7000” or lejguar or prefill or galacto?oligosaccharide* or “galactose oligomer” or oligogalactose or GOS or fructo?oligosaccharide* or Idolax or “Raftilose P95” or neosugar or oligofructose or Metamucil or Plantaglucide or Ispaghul* or (Plantago adj Seed*) or Iso?gel or Reguval or agiocur or arcolax or betajel or fybogel or konsyl or metamucil or mucilax or mucilose or mucofalk or “plantaginis semen” or plantaglucid* or “plantago ovata extract” or “plantago ovata seed” or psyllium or regulan or transilane or “vi siblin” or volcolon or Flax* or Linum* or Lin?seed*).ti,ab,kw. (79488)
5. exp Weight Loss/(46792)
6. ((weight or BMI) adj3 (los* or decrea* or reduc* or watching)).ti,ab,kw. (161745)
7. 1 or 2 (494979)
8. 3 or 4 (100446)
9. 5 or 6 (175368)
10. 7 and 8 and 9 (817)
11. exp animals/not exp humans/(4991746)
12. 10 not 11 (665)

Search Strings for Embase via Elsevier covering results from 1947 to 13 April 2022.

#14 #12 NOT #13 1202
#13 ‘conference abstract’/it 4374972
#12 #10 NOT #11 1450
#11 ‘animals’/exp NOT ‘humans’/exp 5764029
#10 #7 AND #8 AND #9 1709
#9 #5 OR #6 322875
#8 #3 OR #4 196845
#7 #1 OR #2 809870
#6 ((weight OR bmi) NEAR/3 (los* OR decrea* OR reduc* OR watching)):ti,ab,kw 249922
#5 ‘weight loss’/exp 210306
#4 ((diet* NEAR/3 fib*):ti,ab,kw) OR (wheat:ti,ab,kw AND near:ti,ab,kw AND bran*:ti,ab,kw) OR roughage*:ti,ab,kw OR ‘alimentary fib*’:ti,ab,kw OR opti?fib*:ti,ab,kw OR ‘stimulance multi fib*’:ti,ab,kw OR alant*:ti,ab,kw OR dahlin:ti,ab,kw OR inulin*:ti,ab,kw OR synanthrin:ti,ab,kw OR xylan*:ti,ab,kw OR arabinoxylan*:ti,ab,kw OR xyloarabinan*:ti,ab,kw OR hemixylan*:ti,ab,kw OR (beta:ti,ab,kw AND glucan*:ti,ab,kw) OR ‘beta dextroglucan’:ti,ab,kw OR macrogard:ti,ab,kw OR pectin*:ti,ab,kw OR methoxy?pectin:ti,ab,kw OR guar*:ti,ab,kw OR glucotard:ti,ab,kw OR ‘gu-052’:ti,ab,kw OR slocose:ti,ab,kw OR supercol:ti,ab,kw OR ‘cal-ban 3000’:ti,ab,kw OR ‘burtonite v 7 e’:ti,ab,kw OR ‘cyamopsis gum’:ti,ab,kw OR decorpa:ti,ab,kw OR fibraguar:ti,ab,kw OR galactasol:ti,ab,kw OR ‘gum cyamopsis’:ti,ab,kw OR ‘hepart hp 7000’:ti,ab,kw OR lejguar:ti,ab,kw OR prefill:ti,ab,kw OR galacto?oligosaccharide*:ti,ab,kw OR ‘galactose oligomer’:ti,ab,kw OR oligogalactose:ti,ab,kw OR gos:ti,ab,kw OR fructo?oligosaccharide*:ti,ab,kw OR idolax:ti,ab,kw OR ‘raftilose p95’:ti,ab,kw OR neosugar:ti,ab,kw OR oligofructose:ti,ab,kw OR plantaglucid*:ti,ab,kw OR ispaghul*:ti,ab,kw OR (plantago:ti,ab,kw AND near:ti,ab,kw AND seed:ti,ab,kw) OR ‘seed plantago’:ti,ab,kw OR iso?gel:ti,ab,kw OR reguval:ti,ab,kw OR agiocur:ti,ab,kw OR arcolax:ti,ab,kw OR betajel:ti,ab,kw OR fybogel:ti,ab,kw OR konsyl:ti,ab,kw OR metamucil:ti,ab,kw OR mucilax:ti,ab,kw OR mucilose:ti,ab,kw OR mucofalk:ti,ab,kw OR ‘plantaginis semen’:ti,ab,kw OR plantaglucide:ti,ab,kw OR ‘plantago ovata extract’:ti,ab,kw OR ‘plantago ovata seed’:ti,ab,kw OR psyllium:ti,ab,kw OR regulan:ti,ab,kw OR transilane:ti,ab,kw OR ‘vi siblin’:ti,ab,kw OR volcolon:ti,ab,kw OR flax*:ti,ab,kw OR linum*:ti,ab,kw OR lin?seed*:ti,ab,kw 164504
#3 ‘dietary fibre’/exp OR ‘inulin’/exp OR ‘xylans’/exp OR ‘beta-glucans’/exp OR ‘pectins’/exp OR ‘4-galactooligosaccharide’ OR ‘fructooligosaccharide’/exp OR ‘psyllium’/exp OR ‘flax’/exp 67816
#2 adipos*:ti,ab,kw OR obes*:ti,ab,kw OR ((excess NEAR/3 weight):ti,ab,kw) OR overweight:ti,ab,kw 657886
#1 ‘adiposity’/de OR ‘obesity’/exp 601338

Search Strings for Google Scholar covering results until 14 April 2022.

Obesity|Overweight “Dietary fiber”|Soluble fiber” “Body weight” RCT|”randomized controlled trial”.

## Appendix B

**Table A1:** Estimation of evidence certainty according to GRADE [49].

Outcome	Certainty Assessment							Number of Patients		Absolute Effect (95% CI)	Certainty
	Number of Cohorts	Study Design	Risk of Bias	Inconsistency	Indirectness	Imprecision	Other Considerations	Dietary Fibre	Placebo or No Intervention		
Body weight [kg]	**27**	randomised trials	not serious ^a^	not serious ^b^	not serious	not serious ^c^	none	758	670	MD 1.25 SD lower (2.24 lower to 0.25 lower)	High

CI: confidence interval; MD: Mean difference; ^a^ Out of 27 cohorts, 11 (40.7%) qualified for a low risk of bias and only two cohorts showed a high risk of bias due to the randomisation process. ^b^ No significant heterogeneity (I^2^ = 0%, τ^2^ = 0, χ^2^ = 22.00, *p* = 0.69). ^c^ Number participants (*n* = 1428) higher than “optimal information size” (OIS).

## References

[1] World Health Organization Obesity and Overweight. https://www.who.int/news-room/fact-sheets/detail/obesity-and-overweight.

[2] Blüher M. (2019). Obesity: Global epidemiology and pathogenesis. Nat. Rev. Endocrinol..

[3] Waters H., Graf M. (2018). America’s obesity crisis. The Health and Economic Costs of Excess Weight.

[4] National Clinical Guideline Centre (UK) (2014). Obesity: Identification, Assessment and Management of Overweight and Obesity in Children, Young People and Adults: Partial Update of CG43.

[5] Varady K.A., Cienfuegos S., Ezpeleta M., Gabel K. (2022). Clinical application of intermittent fasting for weight loss: Progress and future directions. Nat. Rev. Endocrinol..

[6] Dent R., McPherson R., Harper M.E. (2020). Factors affecting weight loss variability in obesity. Metabolism.

[7] Schlesinger S., Neuenschwander M., Schwedhelm C., Hoffmann G., Bechthold A., Boeing H., Schwingshackl L. (2019). Food Groups and Risk of Overweight, Obesity, and Weight Gain: A Systematic Review and Dose-Response Meta-Analysis of Prospective Studies. Adv. Nutr..

[8] Reynolds A.N., Akerman A.P., Mann J. (2020). Dietary fibre and whole grains in diabetes management: Systematic review and meta-analyses. PLoS Med..

[9] Tang G., Wang D., Long J., Yang F., Si L. (2015). Meta-analysis of the association between whole grain intake and coronary heart disease risk. Am. J. Cardiol..

[10] Trowell H. (1972). Crude fibre, dietary fibre and atherosclerosis. Atherosclerosis.

[11] World Health Organization (2003). Diet, Nutrition, and the Pprevention of Chronic Diseases: Report of a Joint WHO/FAO Expert Consultation.

[12] EFSA Panel on Dietetic Products, Nutrition, and Allergies (NDA) (2010). Scientific opinion on dietary reference values for carbohydrates and dietary fibre. EFSA J..

[13] DACH (2021). Referenzwerte für die Nährstoffzufuhr, Gesellschaften für Ernährung in Deutschland (DGE), Österreich (ÖGE) und der Schweiz (SGE).

[14] Nordic Council of Ministers (2014). Nordic Nutrition Recommendations 2012: Integrating Nutrition and Physical Activity.

[15] U.S. Department of Agriculture, U.S. Department of Health and Human Services (2020). Dietary Guidelines for Americans, 2020–2025.

[16] National Health and Medical Research Council, Australian Government Department of Health and Ageing, New Zealand Ministry of Health (2006). Nutrient Reference Values for Australia and New Zealand: Including Recommended Dietary Intakes.

[17] Institute of Medicine (2005). Dietary Reference Intakes for Energy, Carbohydrate, Fiber, Fat, Fatty Acids, Cholesterol, Protein, and Amino Acids.

[18] Stephen A.M., Champ M.M., Cloran S.J., Fleith M., van Lieshout L., Mejborn H., Burley V.J. (2017). Dietary fibre in Europe: Current state of knowledge on definitions, sources, recommendations, intakes and relationships to health. Nutr. Res. Rev..

[19] Balmer M.L., Ma E.H., Bantug G.R., Grählert J., Pfister S., Glatter T., Jauch A., Dimeloe S., Slack E., Dehio P. (2016). Memory CD8(+) T Cells Require Increased Concentrations of Acetate Induced by Stress for Optimal Function. Immunity.

[20] Balmer M.L., Ma E.H., Thompson A.J., Epple R., Unterstab G., Lötscher J., Dehio P., Schürch C.M., Warncke J.D., Perrin G. (2020). Memory CD8(+) T Cells Balance Pro- and Anti-inflammatory Activity by Reprogramming Cellular Acetate Handling at Sites of Infection. Cell Metab..

[21] Koh A., de Vadder F., Kovatcheva-Datchary P., Bäckhed F. (2016). From Dietary Fiber to Host Physiology: Short-Chain Fatty Acids as Key Bacterial Metabolites. Cell.

[22] Turnbaugh P.J., Hamady M., Yatsunenko T., Cantarel B.L., Duncan A., Ley R.E., Sogin M.L., Jones W.J., Roe B.A., Affourtit J.P. (2009). A core gut microbiome in obese and lean twins. Nature.

[23] Le Chatelier E., Nielsen T., Qin J., Prifti E., Hildebrand F., Falony G., Almeida M., Arumugam M., Batto J.M., Kennedy S. (2013). Richness of human gut microbiome correlates with metabolic markers. Nature.

[24] Overby H.B., Ferguson J.F. (2021). Gut Microbiota-Derived Short-Chain Fatty Acids Facilitate Microbiota: Host Cross Talk and Modulate Obesity and Hypertension. Curr. Hypertens. Rep..

[25] Ho H.V., Sievenpiper J.L., Zurbau A., Blanco Mejia S., Jovanovski E., Au-Yeung F., Jenkins A.L., Vuksan V. (2016). The effect of oat β-glucan on LDL-cholesterol, non-HDL-cholesterol and apoB for CVD risk reduction: A systematic review and meta-analysis of randomised-controlled trials. Br. J. Nutr..

[26] Ho H.V.T., Jovanovski E., Zurbau A., Blanco Mejia S., Sievenpiper J.L., Au-Yeung F., Jenkins A.L., Duvnjak L., Leiter L., Vuksan V. (2017). A systematic review and meta-analysis of randomized controlled trials of the effect of konjac glucomannan, a viscous soluble fiber, on LDL cholesterol and the new lipid targets non-HDL cholesterol and apolipoprotein B. Am. J. Clin. Nutr..

[27] Khan K., Jovanovski E., Ho H.V.T., Marques A.C.R., Zurbau A., Mejia S.B., Sievenpiper J.L., Vuksan V. (2018). The effect of viscous soluble fiber on blood pressure: A systematic review and meta-analysis of randomized controlled trials. Nutr. Metab. Cardiovasc. Dis..

[28] Thompson S.V., Hannon B.A., An R., Holscher H.D. (2017). Effects of isolated soluble fiber supplementation on body weight, glycemia, and insulinemia in adults with overweight and obesity: A systematic review and meta-analysis of randomized controlled trials. Am. J. Clin. Nutr..

[29] Jovanovski E., Mazhar N., Komishon A., Khayyat R., Li D., Blanco Mejia S., Khan T., Jenkins A.L., Smircic-Duvnjak L., Sievenpiper J.L. (2020). Can dietary viscous fiber affect body weight independently of an energy-restrictive diet? A systematic review and meta-analysis of randomized controlled trials. Am. J. Clin. Nutr..

[30] McGill C.R., Fulgoni V.L., Devareddy L. (2015). Ten-year trends in fiber and whole grain intakes and food sources for the United States population: National Health and Nutrition Examination Survey 2001–2010. Nutrients.

[31] Dreher M.L., Davenport A.J. (2013). Hass avocado composition and potential health effects. Crit. Rev. Food Sci. Nutr..

[32] Slavin J.L., Lloyd B. (2012). Health benefits of fruits and vegetables. Adv. Nutr..

[33] National Clinical Guideline Centre (UK) (2016). Obesity in Adults: Prevention and Lifestyle Wweight Management Programmes.

[34] Leeming E.R., Johnson A.J., Spector T.D., Le Roy C.I. (2019). Effect of Diet on the Gut Microbiota: Rethinking Intervention Duration. Nutrients.

[35] Page M.J., McKenzie J.E., Bossuyt P.M., Boutron I., Hoffmann T.C., Mulrow C.D., Shamseer L., Tetzlaff J.M., Akl E.A., Brennan S.E. (2021). The PRISMA 2020 statement: An updated guideline for reporting systematic reviews. BMJ.

[36] Higgins J.P., Thomas J., Chandler J., Cumpston M., Li T., Page M.J., Welch V.A. (2019). Cochrane Handbook for Systematic Reviews of Interventions.

[37] Cooper H. (2015). Research Synthesis and Meta-Analysis: A Step-by-Step Approach.

[38] Matthews D.R., Hosker J.P., Rudenski A.S., Naylor B.A., Treacher D.F., Turner R.C. (1985). Homeostasis model assessment: Insulin resistance and beta-cell function from fasting plasma glucose and insulin concentrations in man. Diabetologia.

[39] Sterne J.A., Savović J., Page M.J., Elbers R.G., Blencowe N.S., Boutron I., Cates C.J., Cheng H.-Y., Corbett M.S., Eldridge S.M. (2019). RoB 2: A revised tool for assessing risk of bias in randomised trials. BMJ.

[40] Viechtbauer W. (2010). Conducting meta-analyses in R with the metafor package. J. Stat. Softw..

[41] R Core Team (2021). R: A Language and Environment for Statistical Computing.

[42] Hedges L.V. (1992). Meta-analysis. J. Educ. Stat..

[43] Hedges L.V. (1983). A random effects model for effect sizes. Psychol. Bull..

[44] Cochran W.G. (1954). The combination of estimates from different experiments. Biometrics.

[45] Higgins J.P., Thompson S.G., Deeks J.J., Altman D.G. (2003). Measuring inconsistency in meta-analyses. BMJ.

[46] Egger M., Smith G.D., Schneider M., Minder C. (1997). Bias in meta-analysis detected by a simple, graphical test. BMJ.

[47] Begg C.B., Mazumdar M. (1994). Operating characteristics of a rank correlation test for publication bias. Biometrics.

[48] Baujat B., Mahé C., Pignon J.P., Hill C. (2002). A graphical method for exploring heterogeneity in meta-analyses: Application to a meta-analysis of 65 trials. Stat. Med..

[49] Schünemann H.B.J., Guyatt G., Oxman A. (2013). GRADE Handbook for Grading Quality of Evidence and Strength of Recommendations.

[50] GRADEpro GDT. GRADEpro Guideline Development Tool [Software]: McMaster University and Evidence Prime, Canada..

[51] Cicero A.F.G., Derosa G., Bove M., Imola F., Borghi C., Gaddi A.V. (2010). Psyllium improves dyslipidaemia, hyperglycaemia and hypertension, while guar gum reduces body weight more rapidly in patients affected by metabolic syndrome following an AHA Step 2 diet. Mediterr. J. Nutr. Metab..

[52] Bomhof M.R., Parnell J.A., Ramay H.R., Crotty P., Rioux K.P., Probert C.S., Jayakumar S., Raman M., Reimer R.A. (2019). Histological improvement of non-alcoholic steatohepatitis with a prebiotic: A pilot clinical trial. Eur. J. Nutr..

[53] Bongartz U., Hochmann U., Grube B., Uebelhack R., Alt F., Erlenbeck C., Peng L.V., Chong P.W., de Costa P. (2022). Flaxseed Mucilage (IQP-LU-104) Reduces Body Weight in Overweight and Moderately Obese Individuals in a 12-Week, Three-Arm, Double-Blind, Randomized and Placebo-Controlled Clinical Study. Obes. Facts.

[54] Calikoglu F., Barbaros U., Uzum A.K., Tutuncu Y., Satman I. (2021). The Metabolic Effects of Pre-probiotic Supplementation After Roux-en-Y Gastric Bypass (RYGB) Surgery: A Prospective, Randomized Controlled Study. Obes. Surg..

[55] Dewulf E.M., Cani P.D., Claus S.P., Fuentes S., Puylaert P.G., Neyrinck A.M., Bindels L.B., de Vos W.M., Gibson G.R., Thissen J.-P. (2013). Insight into the prebiotic concept: Lessons from an exploratory, double blind intervention study with inulin-type fructans in obese women. Gut.

[56] Genta S., Cabrera W., Habib N., Pons J., Carillo I.M., Grau A., Sanchez S. (2009). Yacon syrup: Beneficial effects on obesity and insulin resistance in humans. Clin. Nutr..

[57] Grube B., Chong P.W., Lau K.Z., Orzechowski H.D. (2013). A natural fiber complex reduces body weight in the overweight and obese: A double-blind, randomized, placebo-controlled study. Obesity.

[58] Grunberger G., Jen K.L.C., Artiss J.D. (2007). The benefits of early intervention in obese diabetic patients with FBCx^™^—A new dietary fibre. Diabetes/Metab. Res. Rev..

[59] Guérin-Deremaux L., Li S., Pochat M., Wils D., Mubasher M., Reifer C., Miller L.E. (2011). Effects of NUTRIOSE^®^ dietary fiber supplementation on body weight, body composition, energy intake, and hunger in overweight men. Int. J. Food Sci. Nutr..

[60] Li S., Guerin-Deremaux L., Pochat M., Wils D., Reifer C., Miller L.E. (2010). NUTRIOSE dietary fiber supplementation improves insulin resistance and determinants of metabolic syndrome in overweight men: A double-blind, randomized, placebo-controlled study. Appl. Physiol. Nutr. Metab..

[61] Hassan O.M.S., di Folco U., Nardone M.R., Tubili F., Tubili C. (2020). Fiber enrichment of pasta: Metabolic effects and diet adherence in obese subjects. Mediterr. J. Nutr. Metab..

[62] Hess A.L., Benítez-Páez A., Blædel T., Larsen L.H., Iglesias J.R., Madera C., Sanz Y., Larsen T.M. (2020). The effect of inulin and resistant maltodextrin on weight loss during energy restriction: A randomised, placebo-controlled, double-blinded intervention. Eur. J. Nutr..

[63] Benítez-Páez A., Hess A.L., Krautbauer S., Liebisch G., Christensen L., Hjorth M.F., Larsen T.M., Sanz Y. (2021). Sex, Food, and the Gut Microbiota: Disparate Response to Caloric Restriction Diet with Fiber Supplementation in Women and Men. Mol. Nutr. Food Res..

[64] Georg Jensen M., Kristensen M., Astrup A. (2012). Effect of alginate supplementation on weight loss in obese subjects completing a 12-wk energy-restricted diet: A randomized controlled trial. Am. J. Clin. Nutr..

[65] Kristensen M., Juul S.R., Sorensen K.V., Lorenzen J.K., Astrup A. (2017). Supplementation with dairy calcium and/or flaxseed fibers in conjunction with orlistat augments fecal fat excretion without altering ratings of gastrointestinal comfort. Nutr. Metab..

[66] Pal S., Ho S., Gahler R.J., Wood S. (2016). Effect on body weight and composition in overweight/obese Australian adults over 12 months consumption of two different types of fibre supplementation in a randomized trial. Nutr. Metab..

[67] Parnell J.A., Reimer R.A. (2009). Weight loss during oligofructose supplementation is associated with decreased ghrelin and increased peptide YY in overweight and obese adults. Am. J. Clin. Nutr..

[68] Parnell J.A., Klancic T., Reimer R.A. (2017). Oligofructose decreases serum lipopolysaccharide and plasminogen activator inhibitor-1 in adults with overweight/obesity. Obesity.

[69] Pol K., Christensen R., Bartels E.M., Raben A., Tetens I., Kristensen M. (2013). Whole grain and body weight changes in apparently healthy adults: A systematic review and meta-analysis of randomized controlled studies. Am. J. Clin. Nutr..

[70] Reimer R.A., Wharton S., Green T.J., Manjoo P., Ramay H.R., Lyon M.R., Gahler R.J., Wood S. (2021). Effect of a functional fibre supplement on glycemic control when added to a year-long medically supervised weight management program in adults with type 2 diabetes. Eur. J. Nutr..

[71] Reimer R.A., Willis H.J., Tunnicliffe J.M., Park H., Madsen K.L., Soto-Vaca A. (2017). Inulin-type fructans and whey protein both modulate appetite but only fructans alter gut microbiota in adults with overweight/obesity: A randomized controlled trial. Mol. Nutr. Food Res..

[72] Reimer R.A., Yamaguchi H., Eller L.K., Lyon M.R., Gahler R.J., Kacinik V., Juneja P., Wood S. (2013). Changes in visceral adiposity and serum cholesterol with a novel viscous polysaccharide in Japanese adults with abdominal obesity. Obesity.

[73] Solah V.A., Kerr D.A., Hunt W.J., Johnson S.K., Boushey C.J., Delp E.J., Meng X., Gahler R.J., James A.P., Mukhtar A.S. (2017). Effect of Fibre Supplementation on Body Weight and Composition, Frequency of Eating and Dietary Choice in Overweight Individuals. Nutrients.

[74] Tovar A.R., Caamano Mdel C., Garcia-Padilla S., Garcia O.P., Duarte M.A., Rosado J.L. (2012). The inclusion of a partial meal replacement with or without inulin to a calorie restricted diet contributes to reach recommended intakes of micronutrients and decrease plasma triglycerides: A randomized clinical trial in obese Mexican women. Nutr. J..

[75] Wood R.J., Volek J.S., Davis S.R., Dell’Ova C., Fernandez M.L. (2006). Effects of a carbohydrate-restricted diet on emerging plasma markers for cardiovascular disease. Nutr. Metab..

[76] Wood R.J., Fernandez M.L., Sharman M.J., Silvestre R., Greene C.M., Zern T.L., Shrestha S., Judelson D.A., Gomez A.L., Kraemer W.J. (2007). Effects of a carbohydrate-restricted diet with and without supplemental soluble fiber on plasma low-density lipoprotein cholesterol and other clinical markers of cardiovascular risk. Metabolism.

[77] Cohen J. (1988). Statistical Power Analysis for the Behavioral Sciences.

[78] Magkos F., Fraterrigo G., Yoshino J., Luecking C., Kirbach K., Kelly S.C., de Las Fuentes L., He S., Okunade A.L., Patterson B.W. (2016). Effects of Moderate and Subsequent Progressive Weight Loss on Metabolic Function and Adipose Tissue Biology in Humans with Obesity. Cell Metab..

[79] Warkentin L.M., Majumdar S.R., Johnson J.A., Agborsangaya C.B., Rueda-Clausen C.F., Sharma A.M., Klarenbach S.W., Karmali S., Birch D.W., Padwal R.S. (2014). Weight loss required by the severely obese to achieve clinically important differences in health-related quality of life: Two-year prospective cohort study. BMC Med..

[80] Jensen M.D., Ryan D.H., Apovian C.M., Ard J.D., Comuzzie A.G., Donato K.A., Hu F.B., Hubbard V.S., Jakicic J.M., Kushner R.F. (2014). 2013 AHA/ACC/TOS guideline for the management of overweight and obesity in adults: A report of the American College of Cardiology/American Heart Association Task Force on Practice Guidelines and The Obesity Society. Circulation.

[81] Hamman R.F., Wing R.R., Edelstein S.L., Lachin J.M., Bray G.A., Delahanty L., Hoskin M., Kriska A.M., Mayer-Davis E.J., Pi-Sunyer X. (2006). Effect of weight loss with lifestyle intervention on risk of diabetes. Diabetes Care.

[82] Koutoukidis D.A., Jebb S.A., Zimmerman M., Otunla A., Henry J.A., Ferrey A., Schofield E., Kinton J., Aveyard P., Marchesi J.R. (2022). The association of weight loss with changes in the gut microbiota diversity, composition, and intestinal permeability: A systematic review and meta-analysis. Gut Microbes.

[83] Tilg H., Zmora N., Adolph T.E., Elinav E. (2020). The intestinal microbiota fuelling metabolic inflammation. Nat. Rev. Immunol..

[84] Shaw K., Gennat H., O’Rourke P., del Mar C. (2006). Exercise for overweight or obesity. Cochrane Database Syst. Rev..

[85] Franz M.J., VanWormer J.J., Crain A.L., Boucher J.L., Histon T., Caplan W., Bowman J.D., Pronk N.P. (2007). Weight-loss outcomes: A systematic review and meta-analysis of weight-loss clinical trials with a minimum 1-year follow-up. J. Am. Diet. Assoc..

[86] Khera R., Murad M.H., Chandar A.K., Dulai P.S., Wang Z., Prokop L.J., Loomba R., Camilleri M., Singh S. (2016). Association of Pharmacological Treatments for Obesity with Weight Loss and Adverse Events: A Systematic Review and Meta-analysis. JAMA.

[87] Gloy V.L., Briel M., Bhatt D.L., Kashyap S.R., Schauer P.R., Mingrone G., Bucher H.C., Nordmann A.J. (2013). Bariatric surgery versus non-surgical treatment for obesity: A systematic review and meta-analysis of randomised controlled trials. BMJ.

[88] Deehan E.C., Duar R.M., Armet A.M., Perez-Muñoz M.E., Jin M., Walter J. (2017). Modulation of the gastrointestinal microbiome with nondigestible fermentable carbohydrates to improve human health. Microbiol. Spectr..

[89] Venkataraman A., Sieber J.R., Schmidt A.W., Waldron C., Theis K.R., Schmidt T.M. (2016). Variable responses of human microbiomes to dietary supplementation with resistant starch. Microbiome.

[90] Walker A.W., Ince J., Duncan S.H., Webster L.M., Holtrop G., Ze X., Brown D., Stares M.D., Scott P., Bergerat A. (2011). Dominant and diet-responsive groups of bacteria within the human colonic microbiota. ISME J..

[91] Martínez I., Kim J., Duffy P.R., Schlegel V.L., Walter J. (2010). Resistant starches types 2 and 4 have differential effects on the composition of the fecal microbiota in human subjects. PLoS ONE.

[92] Lozupone C.A., Stombaugh J.I., Gordon J.I., Jansson J.K., Knight R. (2012). Diversity, stability and resilience of the human gut microbiota. Nature.

[93] Xiong Y., Miyamoto N., Shibata K., Valasek M.A., Motoike T., Kedzierski R.M., Yanagisawa M. (2004). Short-chain fatty acids stimulate leptin production in adipocytes through the G protein-coupled receptor GPR41. Proc. Natl. Acad. Sci. USA.

[94] Zaibi M.S., Stocker C.J., O’Dowd J., Davies A., Bellahcene M., Cawthorne M.A., Brown A.J., Smith D.M., Arch J.R. (2010). Roles of GPR41 and GPR43 in leptin secretory responses of murine adipocytes to short chain fatty acids. FEBS Lett..

[95] Roager H.M., Vogt J.K., Kristensen M., Hansen L.B.S., Ibrügger S., Mærkedahl R.B., Bahl M.I., Lind M.V., Nielsen R.L., Frøkiær H. (2019). Whole grain-rich diet reduces body weight and systemic low-grade inflammation without inducing major changes of the gut microbiome: A randomised cross-over trial. Gut.

[96] Suhr J., Vuholm S., Iversen K.N., Landberg R., Kristensen M. (2017). Wholegrain rye, but not wholegrain wheat, lowers body weight and fat mass compared with refined wheat: A 6-week randomized study. Eur. J. Clin. Nutr..

[97] Burley V.J., Paul A.W., Blundell J.E. (1993). Influence of a high-fibre food (myco-protein) on appetite: Effects on satiation (within meals) and satiety (following meals). Eur. J. Clin. Nutr..

[98] Wanders A.J., Jonathan M.C., van den Borne J.J., Mars M., Schols H.A., Feskens E.J., de Graaf C. (2013). The effects of bulking, viscous and gel-forming dietary fibres on satiation. Br. J. Nutr..

[99] Odunsi S.T., Vázquez-Roque M.I., Camilleri M., Papathanasopoulos A., Clark M.M., Wodrich L., Lempke M., McKinzie S., Ryks M., Burton D. (2010). Effect of alginate on satiation, appetite, gastric function, and selected gut satiety hormones in overweight and obesity. Obesity.

