# Supplementary material for: Prolonged Isolated Soluble Dietary Fibre Supplementation in Overweight and Obese Patients: A Systematic Review with Meta-Analysis of Randomised Controlled Trials
Source: Nutrients. 2022 Jun 24;14(13):2627. doi: 10.3390/nu14132627 (PMC9268533; doi:10.3390/nu14132627)
Supplement: Supplementary file 1 [file nutrients-14-02627-s001.zip › nutrients-1774386-supplementary.pdf]

Isolated Soluble Dietary Fibre Supplementation in Overweight and Obese Patients: A Systematic Review with Meta-Analysis of Randomised Controlled Trials

Supplementary 1. Excluded Studies

Table S1. Excluded studies according to Figure 1.

Exclusion reason	Number of excluded studies	References of excluded studies
SDF content <50%	12	[1], [2],[3], [4], [5], [6], [7], [8], [9], [10], [11], [12]
Duration <12 weeks	13	[13], [14], [15], [16], [17], [18], [19], [20], [21], [22], [23], [24], [25]
Secondary analysis	1	[26]
Weight maintenance	4	[27], [28], [29], [30]

SDF = soluble dietary fibre.

Supplementary 2. Study Characteristics

Table S2. Study and participant characteristics of the 22 included RCTs.

Author, Year	Intervention, Dose [g/d]	Control compound	Funding	Bodyweight baseline [1] [kg]		Bodyweight final [1] [kg]	
				Intervention	Control	Intervention	Control
Bomhof, 2019 [31]	FOS orally, 8-16 g/day	Maltodextrin	FG	101.3 ± 11.4	102.1 ± 8.1	101.2 ± 12.2	102.3 ± 8.3
Bongartz, 2022 [32]	Flaxseed mucilage powder in water, 5.1 g/day	Standard excipients	IND	85.31 ± 12.56	87.42 ± 12.92	80.35 ± 12.45	86.09 ± 12.65
	Flaxseed mucilage powder in water, 2.6 g/day	Standard excipients	IND	89.47 ± 14.28	85.77 ± 14.23	87.42 ± 12.92	86.09 ± 12.65
Calikoglu, 2021 [33]	Inulin + FOS powder in yogurt, 10 g/day	Yogurt	FG	139.9 ± 20.3	147.5 ± 32.5	99.0 ± 18.8	104.4 ± 21.9
Cicero, 2010 [34]	Guar gum orally, 7 g/day	NT	NR	79.9 ± 4.5	80.1 ± 4.3	78.3 ± 4.4	78.9 ± 4.3
Dewulf, 2013 [35]	Inulin + FOS powder in fluid, 16 g/day	Maltodextrin	FG	99.13 ± 16.25	97.46 ± 15.79	98.20 ± 16.43	96.95 ± 17.14
Genta, 2009 [36]	FOS syrup, 0.14 g/kg BW	Placebo syrup	FG	91.2 ± 8.4	90.7 ± 10.3	76.2 ± 6.1	92.3 ± 10.1
Grube, 2013 [37]	Litramine IQP G-002AS tablets, 3 g/day	Cellulose	IND	113.1 ± 4.2	116.0 ± 3.6	112.7 ± 4.2	116.4 ± 3.6
Grunberger, 2007 [38]	α-Cyclodextrin tablets, 6 g/day	Indigestible starch	NR	85.3 ± 11.8	81.8 ± 11.0	81.5 ± 11.5	80.4 ± 10.5
Guérin-Deremaux, 2011 [39, 40]	Nutriose[2] powder in fruit juice, 29 g/day	Maltodextrin	IND	74.0 ± 3.35	73.9 ± 3.3	72.53 ± 3.36	74.03 ± 3.41
Hassan, 2020 [41]	Inulin + RS pasta, 15 g/100 g pasta	Regular pasta	NR	94.37 ± 8.88	90.71 ± 13.05	88.08 ±10.06	88.79 ± 13.43
Hess, 2020 [42, 43]	Inulin powder in milk, 20 g/day	Maltodextrin	FG	95.4 ± 16.7	103.6 ± 15.9	89.3 ± 16.4	98.1 ± 15.8
Jensen, 2012 [44]	Alginate powder in water, 15 g/day	Maltodextrin and Sucrose	IND	104.30 ± 2.37	99.51 ± 2.20	97.60 ± 2.22	94.47 ± 2.11
Kristensen, 2017 [45]	Flaxseed fibre powder in fluid or food, 5 g/day	Whole grain rice flour	FG, IND	91.2 ± 3.9	98.9 ± 8.4	87.4 ± 3.8	94.0 ± 9.9
	Flaxseed fibre powder in fluid or food, 5 g/day	Whole grain rice flour	FG, IND	95.6 ± 2.8	103.4 ± 5.2	91.4 ± 2.4	98.9 ± 4.9
Pal, 2016 [46]	PGX[3] powder in water, 15 g/day	Rice flour	IND	96.2 ± 2.9	94.7 ± 2.5	NR	NR
Parnell, 2009 [47, 48]	FOS powder in fluid, 21 g/day	Maltodextrin	FG	83.4 ± 13.0	80.2 ± 12.8	82.3 ± 2.6	80.7 ± 3.1
Pol, 2018 [49]	FOS bar, 16 g/day	Control bar	IND	90.9 ± 11.3	90.2 ± 12.6	90.7 ± 11.3	90.6 ± 11.3
Reimer, 2021 [50]	PGX[3] spinkled on food, 15-20 g/day	Rice flour	IND	112.2 ± 25.00	116.3 ± 23.8	106.7 ± 21.7	113.7 ± 28.2
Reimer, 2017 [51]	FOS + Inulin bar, 8 g/day	Control bar	IND	92.4 ± 3.8	90.3 ± 4.8	92.5 ± 3.7	90.7 ± 4.9
	FOS + Inulin protein bar, 8 g/day	Protein bar	IND	96.5 ± 5.2	89.2 ± 3.9	96.4 ± 5.3	88.6 ± 3.8
Reimer, 2013 [52]	PGX[3] powder with yogurt, 15 g/day	Rice flour	IND	71.0 ± 1.3	70.3 ± 1.4	70.9 ± 1.4	70.4 ± 1.4
Solah, 2017 [53]	PGX[3] softgel capsules with water, 8-11 g/day	Rice flour	IND	82.7 ± 16.8	81.3 ± 17.7	83.2 ± 16.8 [4]	81.3 ± 17.7 [4]
	PGX[3] granules in food or fluid, 12 g/day	Rice flour	IND	80.9 ± 16.6	81.3 ± 17.7	80.4 ± 16.6 [4]	81.3 ± 17.7 [4]
Tovar, 2012 [54]	Inulin powder in milk with PMR powder, 10 g/day	PMR powder	IND	75.96 ± 11.71	75.79 ± 12.45	73.68 ± 10.08	71.70 ± 11.63
	Inulin powder in fluid, 10 g/day	NT	IND	76.45 ± 11.07	76.55 ± 10.96	73.55 ± 10.85	73.74 ± 10.57
Wood, 2006 [55, 56]	Glucomannan capsules with water, 3 g/day	Maltodextrin	FG, IND	93.6 ± 12.2	93.0 ± 16.0	86.2 ± 13.2	85.5 ± 15.9

[1] mean ± SD; [2] resistant corn dextrin, [3] combination of glucomannan, sodium alginate, and xanthan gum; [4] SD after 12 weeks estimated according to Cochrane; BW = body weight; FG = federal government/university; FOS = fructooligosaccharides; IND = industry; NR = not reported; PF = private foundation; RS = resistant starch.

Supplementary 3. Forest Plots

Author and Year	Fibre *n*	Control *n*	Weight	SMD 95% CI	SMD Random effects, 95% CI	Risk of Bias A	B	C	D	E	F
Bomhof, 2019	8	6	1.0%	-0.03 [-1.52, 1.46]		+	+	+	+	+	+
Bongartz, 2022 (1)	34	33	4.7%	-0.29 [-0.97, 0.39]		+	+	+	+	+	+
Bongartz, 2022 (2)	33	33	4.7%	-0.15 [-0.83, 0.53]		+	+	+	+	+	+
Calikoglu, 2021	14	16	1.5%	-0.51 [-1.72, 0.70]		+	!	+	+	!	!
Cicero, 2010	46	45	6.3%	-0.08 [-0.67, 0.51]		+	+	+	+	!	!
Dewulf, 2013	15	15	2.1%	-0.03 [-1.05, 0.99]		+	!	!	+	!	!
Genta, 2009	20	15	2.0%	-2.16 [-3.20, -1.12]		+	+	+	+	!	!
Grube, 2013	59	59	8.1%	-0.19 [-0.71, 0.33]		+	+	+	+	+	+
Grunberger, 2007	20	27	3.3%	-0.20 [-1.01, 0.61]		+	+	+	+	!	!
Guérin-Deremaux, 2011	57	57	8.1%	-0.47 [-0.99, 0.05]		+	+	+	+	+	+
Hassan, 2020	15	15	2.1%	-0.50 [-1.52, 0.52]		+	+	+	+	!	!
Hess, 2020	42	44	6.3%	-0.02 [-0.61, 0.57]		+	!	+	+	+	!
Jensen, 2012	38	42	3.0%	-0.57 [-1.42, 0.28]		+	+	+	+	!	!
Kristensen, 2017 (1)	10	6	1.0%	-0.45 [-1.93, 1.03]		-	+	+	+	+	-
Kristensen, 2017 (2)	10	12	1.3%	-0.68 [-1.98, 0.62]		-	+	+	+	+	-
Pal, 2016	32	25		Not estimable		+	+	+	+	+	+
Parnell, 2009	20	17	2.6%	-0.17 [-1.09, 0.75]		+	+	+	+	+	+
Pol, 2018	29	26	3.8%	-0.05 [-0.81, 0.71]		!	!	!	+	+	!
Reimer, 2021	49	43	8.1%	-0.13 [-0.65, 0.39]		+	+	+	+	!	!
Reimer, 2017 (1)	26	27	4.0%	-0.06 [-0.79, 0.67]		+	+	+	+	+	+
Reimer, 2017 (2)	22	21	3.8%	0.13 [-0.63, 0.89]		+	+	+	+	+	+
Reimer, 2013	28	28	4.0%	-0.14 [-0.87, 0.59]		+	!	!	+	!	!
Solah, 2017 (1)	32	19	4.4%	0.03 [-0.68, 0.74]		+	+	+	+	+	+
Solah, 2017 (2)	32	19	4.4%	-0.03 [-0.74, 0.68]		+	+	+	+	+	+
Tovar, 2012 (1)	23	28	3.5%	0.13 [-0.65, 0.91]		+	+	+	+	!	!
Tovar, 2012 (2)	30	29	4.0%	-0.01 [-0.74, 0.72]		+	+	+	+	!	!
Wood, 2006	14	15	2.0%	-0.11 [-1.15, 0.93]		+	+	+	+	!	!
**Total (95% CI)**	**726**	**697**	**100.0%**	**-0.20 [-0.35, -0.05]**							

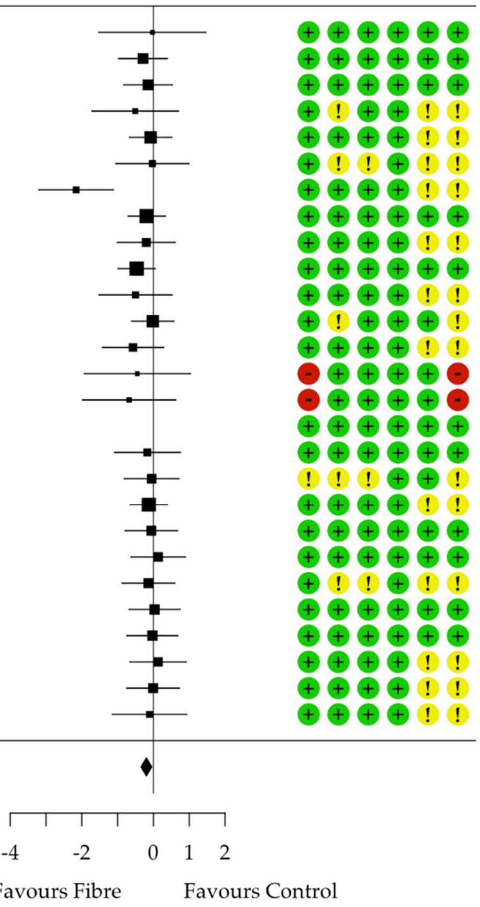

Heterogeneity: $Tau^2 = 0.00$; $Chi^2 = 20.18$, df = 25 ($p = 0.74$); $I^2 = 0\%$
Test for overall effect: $Z = -2.63$ ($p = 0.01$)

Risk of bias legend
(A) Randomisation process
(B) Deviation from intended intervention
(C) Missing outcome data
(D) Measurement of outcome
(E) Selection of reported results
(F) Overall bias

Figure S1. Isolated soluble dietary fibre supplementation significantly decreased body weight [kg] in overweight and obese people (n = 1428). Black rectangles represent SMD for each study; the size of the rectangle is proportional to the weight of the study for the pooled effect. Horizontal line indicates 95% CI. The black diamond summarizes the pooled SMD data. SMD = Standardized mean difference; (1) / (2) indicate cohort 1 and 2 of study.

Author and Year	Fibre *n*	Control *n*	Weight	MD 95% CI
Bomhof, 2019	8	6	1.3%	0.10 [-4.73, 4.93]
Dewulf, 2013	15	15	2.4%	-0.67 [-4.77, 3.43]
Grube, 2013	59	59	7.7%	-0.80 [-26.92, 25.32]
Guérin-Deremaux, 2011	57	57	41.9%	-0.34 [-0.82, 0.14]
Hess, 2020	42	44	6.0%	-0.50 [-4.84, 3.84]
Jensen, 2012	38	42	8.8%	-2.30 [-3.79, -0.81]
Parnell, 2009	20	17	2.6%	-0.60 [-7.75, 6.55]
Pol, 2018	29	26	3.8%	-0.10 [-5.82, 5.62]
Reimer, 2017 (1)	26	27	9.7%	0.00 [-1.13, 1.13]
Reimer, 2017 (2)	22	21	5.8%	1.00 [-0.63, 2.63]
Tovar, 2012 (1)	23	28	3.7%	0.71 [-3.77, 5.19]
Tovar, 2012 (2)	30	29	4.2%	-0.03 [-4.53, 4.47]
Wood, 2006	14	15	2.2%	0.60 [-4.67, 5.87]
**Total (95% CI)**	**383**	**386**	**100.0%**	**-0.37 [-2.46, 1.71]**

Heterogeneity: $Tau^2 = 0.00$; $Chi^2 = 10.00$, df = 12 ($p = 0.62$); $I^2 = 0\%$
Test for overall effect: $Z = -0.35$ ($p = 0.73$)

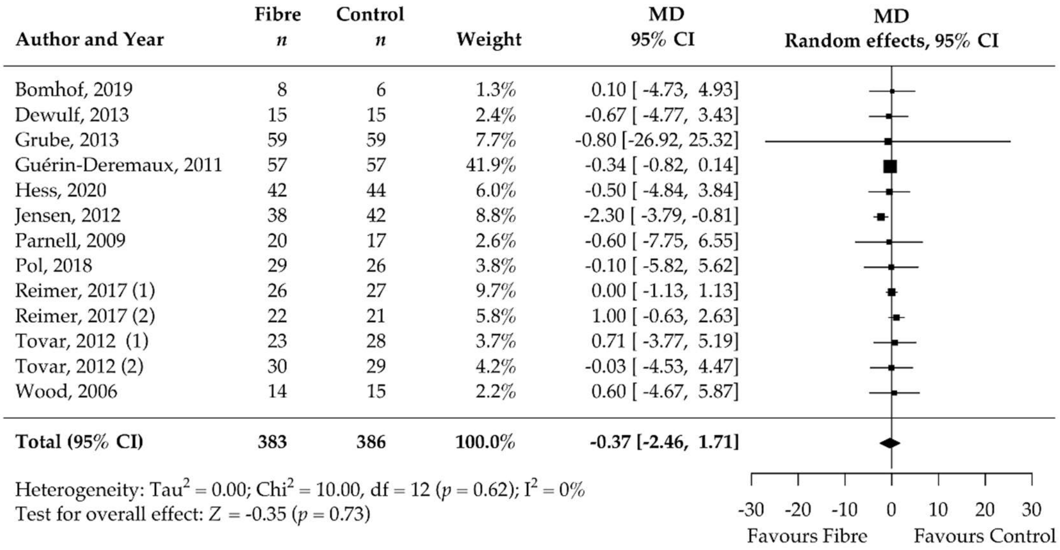

Figure S2: The effect of soluble dietary fibre supplementation on body fat [%] in overweight and obese people. Black rectangles represent MD for each study; the size of the rectangle is proportional to the weight of the study for the pooled effect. Horizontal lines indicate 95% CI. The black diamond summarizes the pooled MD data. MD = Mean difference; (1) / (2) indicate cohort 1 and 2 of study.

Author and Year	Fibre *n*	Control *n*	Weight	MD 95% CI
Bongartz, 2022 (1)	34	33	6.6%	-0.07 [-0.27, 0.13]
Bongartz, 2022 (2)	33	33	6.5%	-0.03 [-0.23, 0.17]
Calikoglu, 2021	14	16	2.4%	0.27 [-0.41, 0.95]
Cicero, 2010	46	45	7.2%	-0.70 [-1.14, -0.26]
Dewulf, 2013	15	15	2.5%	0.03 [-0.49, 0.55]
Grunberger, 2007	20	27	5.1%	0.20 [0.00, 0.40]
Hassan, 2020	15	15	2.8%	-0.41 [-0.75, -0.07]
Hess, 2020	42	44	6.6%	-0.10 [-1.66, 1.46]
Jensen, 2012	38	42	20.8%	-0.11 [-0.17, -0.05]
Kristensen, 2017 (1)	10	6	16.0%	-0.08 [-0.14, -0.02]
Kristensen, 2017 (2)	10	12	16.4%	0.01 [-0.05, 0.07]
Reimer, 2021	49	43	7.2%	-0.30 [-0.85, 0.25]
**Total (95% CI)**	**326**	**331**	**100.0%**	**-0.11 [-0.23, 0.01]**

Heterogeneity: $Tau^2 = 0.00$; $Chi^2 = 28.68$, df = 11 ($p = 0.00$); $I^2 = 0\%$
Test for overall effect: $Z = -1.85$ ($p = 0.06$)

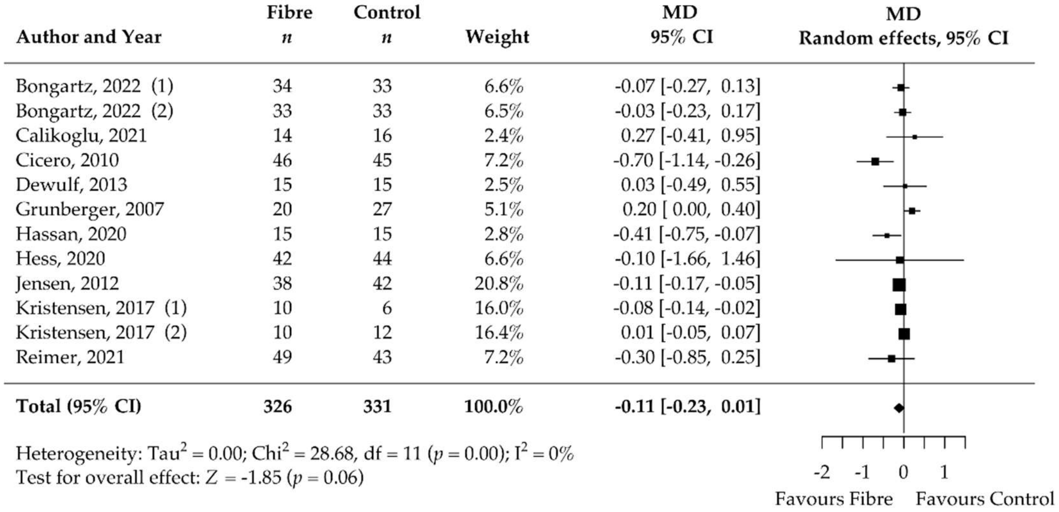

Figure S3: The effect of soluble dietary fibre supplementation on HbA_{1c} [%] in overweight and obese people. Black rectangles represent MD for each study; the size of the rectangle is proportional to the weight of the study for the pooled effect. Horizontal lines indicate 95% CI. The black diamond summarizes the pooled MD data. MD = Mean difference; (1) / (2) indicate cohort 1 and 2 of study.

Author and Year	Fibre n	Control n	Weight	MD 95% CI
Bomhof, 2019	8	6	1.2%	0.36 [-0.62, 1.34]
Calikoglu, 2021	14	16	2.4%	0.34 [-1.17, 1.85]
Cicero, 2010	46	45	7.5%	-0.47 [-1.09, 0.15]
Dewulf, 2013	15	15	2.5%	-0.16 [-1.12, 0.80]
Genta, 2009	20	15	3.1%	-0.28 [-0.83, 0.27]
Hassan, 2020	15	15	2.5%	0.22 [-0.61, 1.05]
Hess, 2020	42	44	7.3%	-0.18 [-0.62, 0.26]
Jensen, 2012	38	42	28.5%	0.10 [0.04, 0.16]
Kristensen, 2017 (1)	10	6	2.4%	0.15 [-0.13, 0.43]
Kristensen, 2017 (2)	10	12	4.0%	-0.23 [-0.43, -0.03]
Reimer, 2013	28	28	26.6%	-0.25 [-0.31, -0.19]
Tovar, 2012 (1)	23	28	4.3%	0.04 [-0.51, 0.59]
Tovar, 2012 (2)	30	29	5.4%	0.13 [-0.21, 0.47]
Wood, 2006	14	15	2.5%	-0.15 [-0.83, 0.53]
**Total (95% CI)**	**313**	**316**	**100.0%**	**-0.08 [-0.17, 0.01]**

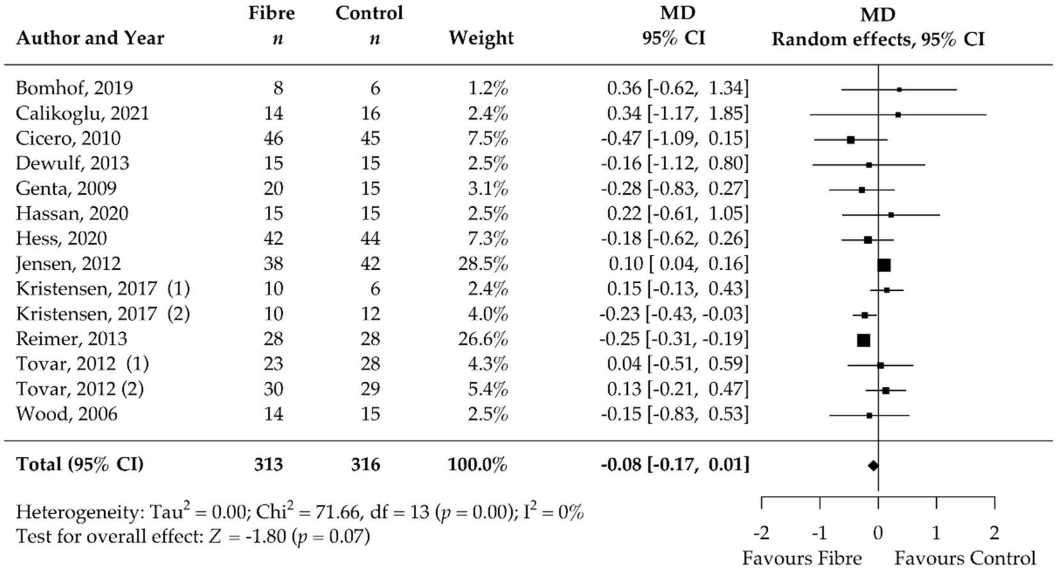

Heterogeneity: $Tau^2 = 0.00$; $Chi^2 = 71.66$, $df = 13$ ($p = 0.00$); $I^2 = 0\%$
Test for overall effect: $Z = -1.80$ ($p = 0.07$)

Figure S4: The effect of soluble dietary fibre supplementation on fasting blood glucose [mmol/L] in overweight and obese people. Black rectangles represent MD for each study; the size of the rectangle is proportional to the weight of the study for the pooled effect. Horizontal lines indicate 95% CI. The black diamond summarizes the pooled MD data. MD = Mean difference; (1) / (2) indicate cohort 1 and 2 of study.

Author and Year	Fibre n	Control n	Weight	MD 95% CI
Dewulf, 2013	15	15	27.9%	-0.05 [-0.49, 0.39]
Grunberger, 2007	20	27	10.2%	1.30 [-0.00, 2.60]
Hess, 2020	42	44	14.1%	0.81 [-3.38, 5.00]
Jensen, 2012	38	42	32.1%	-0.50 [-0.98, -0.02]
Kristensen, 2017 (1)	10	6	3.7%	-0.45 [-2.47, 1.57]
Kristensen, 2017 (2)	10	12	4.6%	-0.77 [-2.80, 1.26]
Wood, 2006	14	15	7.4%	-0.02 [-1.31, 1.27]
**Total (95% CI)**	**149**	**161**	**100.0%**	**0.02 [-0.64, 0.67]**

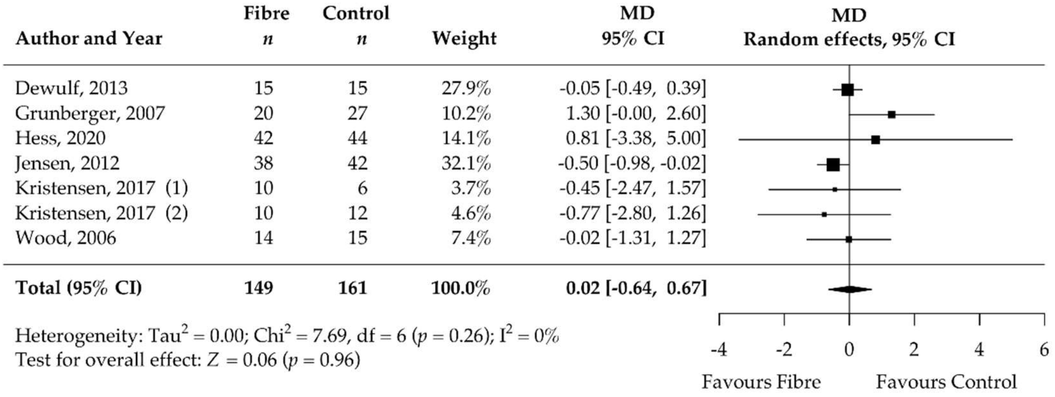

Heterogeneity: $Tau^2 = 0.00$; $Chi^2 = 7.69$, $df = 6$ ($p = 0.26$); $I^2 = 0\%$
Test for overall effect: $Z = 0.06$ ($p = 0.96$)

Figure S5: The effect of soluble dietary fibre supplementation on CRP [mg/L] in overweight and obese people. Black rectangles represent MD for each study; the size of the rectangle is proportional to the weight of the study for the pooled effect. Horizontal lines indicate 95% CI. The black diamond summarizes the pooled MD data. MD = Mean difference; (1) / (2) indicate cohort 1 and 2 of study.

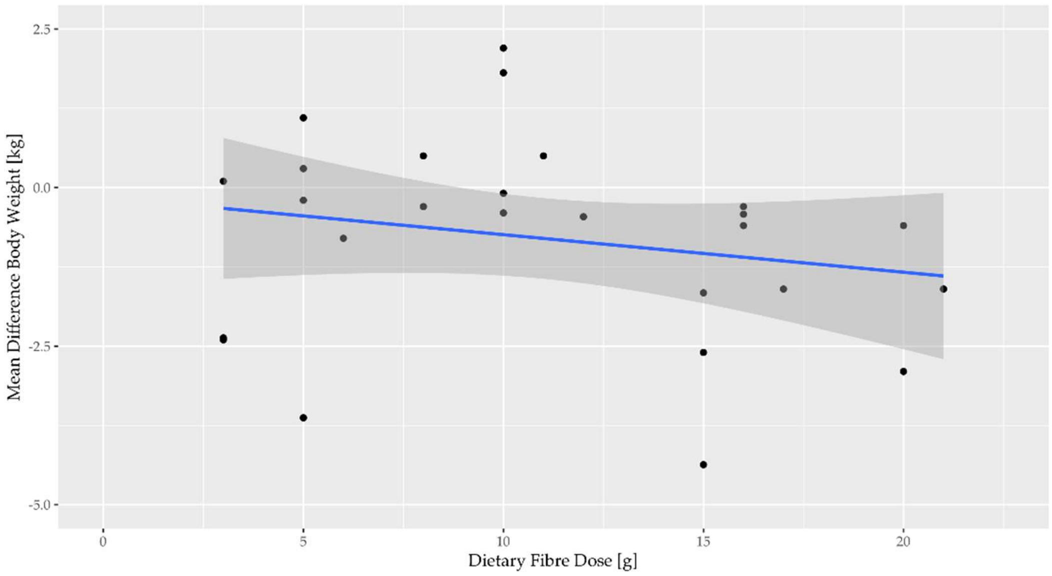

Figure S6. Relationship of supplementation dose and mean difference of body weight.

Supplementary 3. Funnel Plots (Publication Bias)

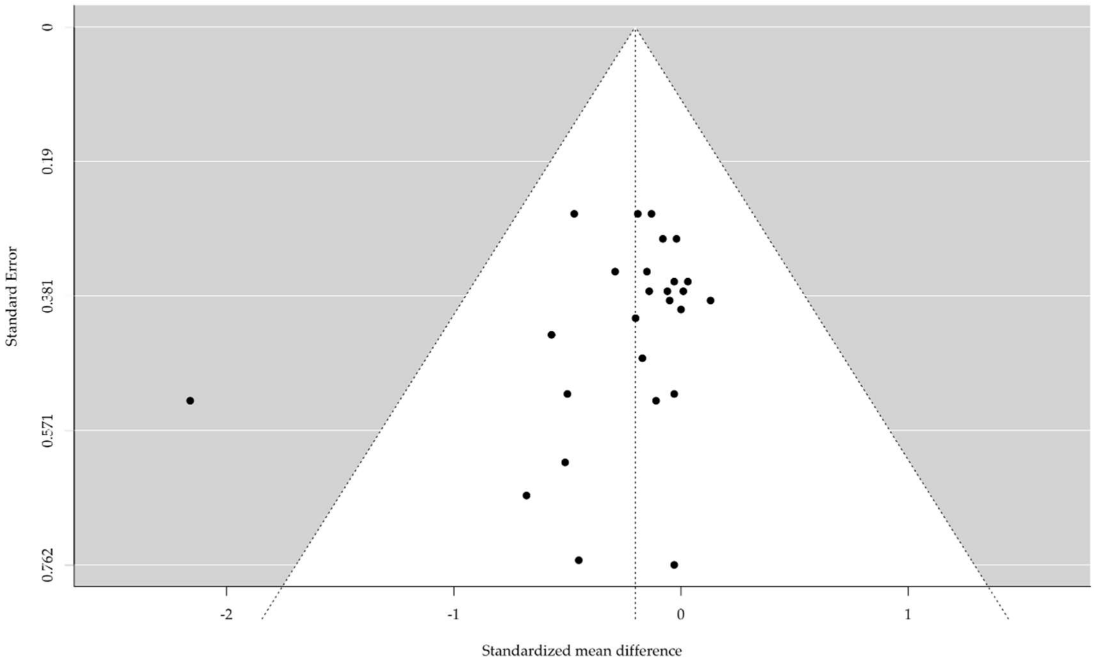

Figure S7. Publication bias analysis with the funnel plot for the primary outcome of body weight.

Supplementary 4. Bajut Plot

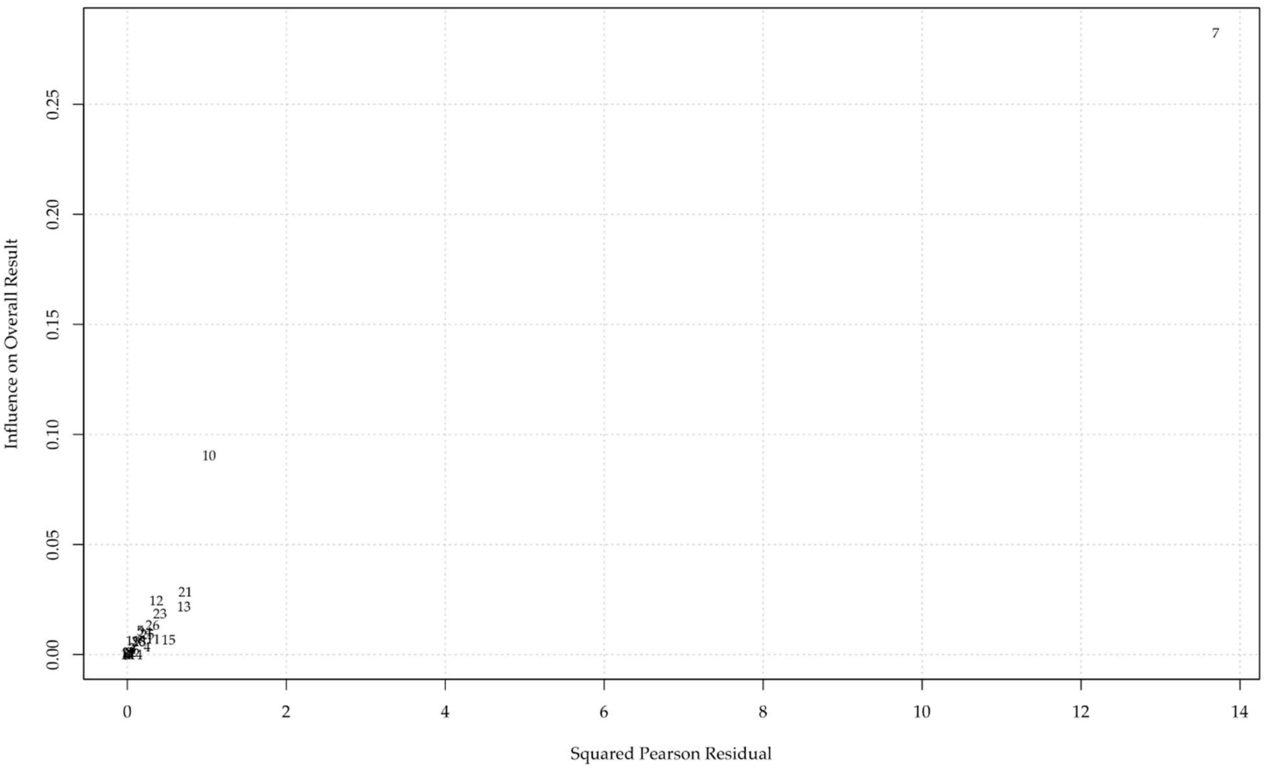

Figure S8: Bajut plot for the outcome body weight.

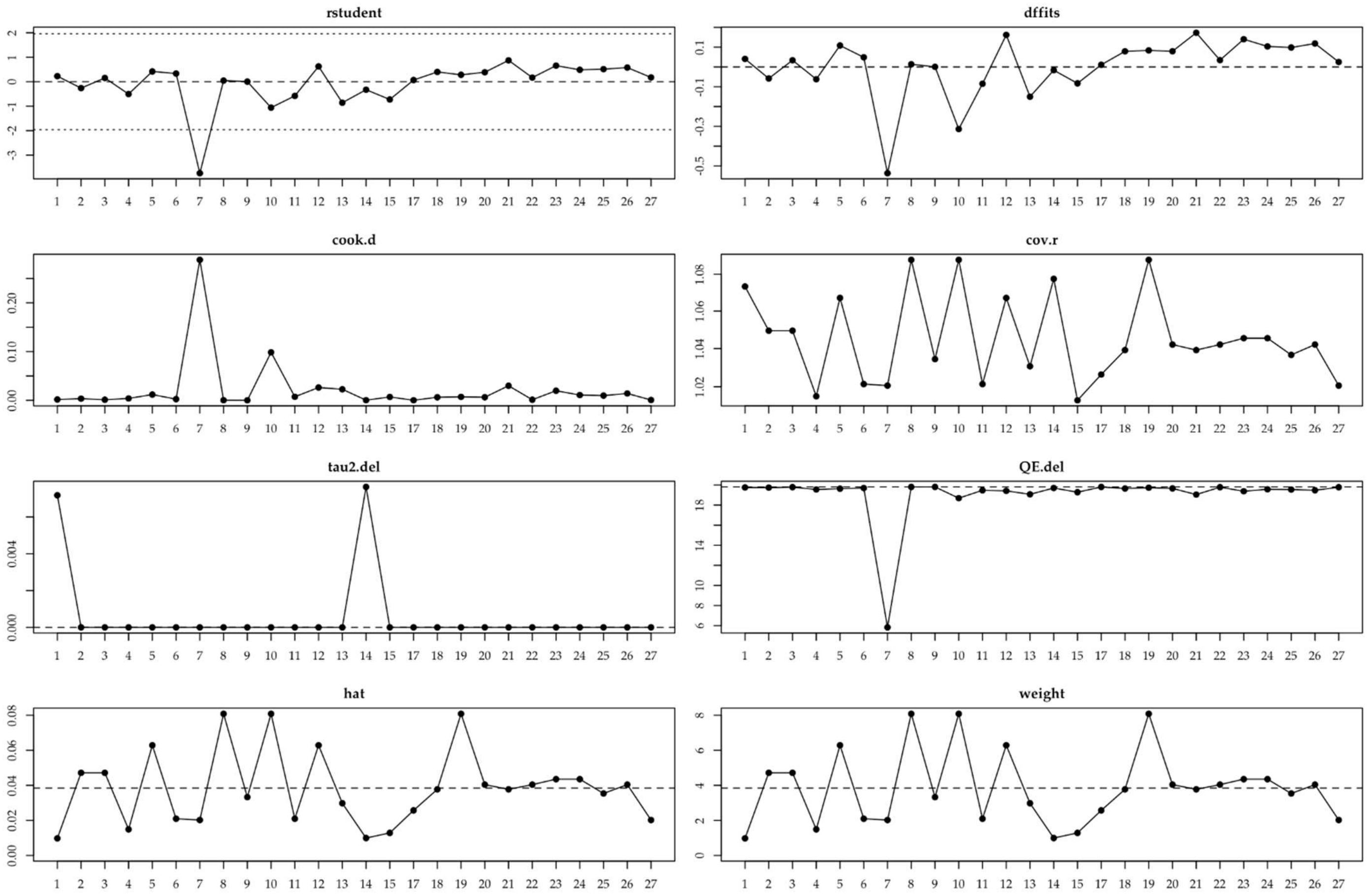

Figure S9: Influential analysis according to the influence function of the metaphor package in R.

References

1. Yari, Z., et al., *Flaxseed Supplementation Improves Anthropometric measurements, Metabolic, and Inflammatory Biomarkers in Overweight and Obese Adults.* International Journal for Vitamin and Nutrition Research, 2020.
2. Chang, H.C., et al., *Oat prevents obesity and abdominal fat distribution, and improves liver function in humans.* Plant Foods for Human Nutrition, 2013. **68**(1): p. 18-23.
3. Kobayakawa, A., et al., *Improvement of fasting plasma glucose level after ingesting moderate amount of dietary fiber in Japanese men with mild hyperglycemia and visceral fat obesity.* Journal of Dietary Supplements, 2013. **10**(2): p. 129-41.
4. Pal, S., et al., *The effect of a fibre supplement compared to a healthy diet on body composition, lipids, glucose, insulin and other metabolic syndrome risk factors in overweight and obese individuals.* British Journal of Nutrition, 2011. **105**(1): p. 90-100.
5. Beck, E.J., et al., *Oat beta-glucan supplementation does not enhance the effectiveness of an energy-restricted diet in overweight women.* British Journal of Nutrition, 2010. **103**(8): p. 1212-22.
6. Salas-Salvadó, J., et al., *Effect of two doses of a mixture of soluble fibres on body weight and metabolic variables in overweight or obese patients: A randomised trial.* British Journal of Nutrition, 2008. **99**(6): p. 1380-1387.
7. Santas, J., E. Lazaro, and J. Cune, *Effect of a polysaccharide-rich hydrolysate from Saccharomyces cerevisiae (LipiGo R) in body weight loss: randomised, double-blind, placebo-controlled clinical trial in overweight and obese adults.* Journal of the Science of Food & Agriculture, 2017. **97**(12): p. 4250-4257.
8. Valero-Pérez, M., et al., *Regular consumption of LIPIGO® promotes the reduction of body weight and improves the rebound effect of obese people undergo a comprehensive weight loss program.* Nutrients, 2020. **12**(7): p. 1-14.
9. Aoe, S., et al., *Effects of high beta-glucan barley on visceral fat obesity in Japanese individuals: A randomized, double-blind study.* Nutrition, 2017. **42**: p. 1-6.
10. Hiel, S., et al., *Link between gut microbiota and health outcomes in inulin -treated obese patients: Lessons from the Food4Gut multicenter randomized placebo-controlled trial.* Clinical Nutrition, 2020. **39**(12): p. 3618-3628.
11. Lambert, J.E., et al., *Consuming yellow pea fiber reduces voluntary energy intake and body fat in overweight/obese adults in a 12-week randomized controlled trial.* Clinical Nutrition, 2017. **36**(1): p. 126-133.
12. Hu, X., et al., *Soy fiber improves weight loss and lipid profile in overweight and obese adults: a randomized controlled trial.* Molecular Nutrition & Food Research, 2013. **57**(12): p. 2147-54.
13. Vaghef-Mehrabany, E., et al., *Calorie restriction in combination with prebiotic supplementation in obese women with depression: effects on metabolic and clinical response.* Nutritional Neuroscience, 2021. **24**(5): p. 339-353.
14. Kuang, X., et al., *Defatted flaxseed flour improves weight loss and lipid profile in overweight and obese adults: a randomized controlled trial.* Food & Function, 2020. **11**(9): p. 8237-8247.
15. Briganti, S., et al., *Effect of an isocaloric diet containing fiber-enriched flour on anthropometric and biochemical parameters in healthy non-obese non-diabetic subjects.* Journal of Clinical Biochemistry & Nutrition, 2015. **57**(3): p. 217-22.
16. Reyna-Villasmil, N., et al., *Oat-derived beta-glucan significantly improves HDLC and diminishes LDLC and non-HDL cholesterol in overweight individuals with mild hypercholesterolemia.* American Journal of Therapeutics, 2007. **14**(2): p. 203-12.
17. Comerford, K.B., et al., *The beneficial effects of alpha-cyclodextrin on blood lipids and weight loss in healthy humans.* Obesity, 2011. **19**(6): p. 1200-4.
18. Leão, L.S.C.D.S., et al., *Addition of oat bran reduces HDL-C and does not potentialize effect of a low-calorie diet on remission of metabolic syndrome: A pragmatic, randomized, controlled, open-label nutritional trial.* Nutrition, 2019. **65**: p. 126-130.
19. Salmean, Y.A., *Using inulin fiber supplementation with MyPlate recommendations promotes greater weight loss in obese women.* Progress in Nutrition, 2019. **21**: p. 81-85.
20. Dall'Alba, V., et al., *Improvement of the metabolic syndrome profile by soluble fibre - guar gum - in patients with type 2 diabetes: a randomised clinical trial.* The British journal of nutrition, 2013. **110**(9): p. 1601-1610.

21. Heini, A.F., et al., *Effect of hydrolyzed guar fiber on fasting and postprandial satiety and satiety hormones: a double-blind, placebo-controlled trial during controlled weight loss.* International Journal of Obesity & Related Metabolic Disorders: Journal of the International Association for the Study of Obesity, 1998. **22**(9): p. 906-9.
22. Malkova, D., et al., *Moderate intensity exercise training combined with inulin-propionate ester supplementation increases whole body resting fat oxidation in overweight women.* Metabolism: Clinical & Experimental, 2020. **104**: p. 154043.
23. Sari-Sarraf, V., R. Amirsasan, and F. Halalkhor, *Effect of concurrent training and flaxseed supplementation on Insulin Indicators and body composition in overweight women.* Iranian Journal of Obstetrics, Gynecology and Infertility, 2018. **21**(8): p. 9-21.
24. Ble-Castillo, J.L., et al., *Effects of native banana starch supplementation on body weight and insulin sensitivity in obese type 2 diabetics.* International Journal of Environmental Research & Public Health [Electronic Resource], 2010. **7**(5): p. 1953-62.
25. Fernandes, R., et al., *Effects of Prebiotic and Synbiotic Supplementation on Inflammatory Markers and Anthropometric Indices After Roux-en-Y Gastric Bypass: A Randomized, Triple-blind, Placebo-controlled Pilot Study.* Journal of Clinical Gastroenterology, 2016. **50**(3): p. 208-17.
26. Nachit, M., et al., *A dynamic association between myosteatosis and liver stiffness: Results from a prospective interventional study in obese patients.* JHEP Reports : Innovation in Hepatology / EASL, 2021. **3**(4): p. 100323.
27. Pasman, W.J., et al., *The effectiveness of long-term fibre supplementation on weight maintenance in weight-reduced women.* International Journal of Obesity & Related Metabolic Disorders: Journal of the International Association for the Study of Obesity, 1997. **21**(7): p. 548-55.
28. Grube, B., et al., *Weight Maintenance with Litramine (IQP-G-002AS): A 24-Week Double-Blind, Randomized, Placebo-Controlled Study.* Journal of Obesity, 2015. **2015**: p. 953138.
29. Guess, N.D., et al., *A randomized controlled trial: the effect of inulin on weight management and ectopic fat in subjects with prediabetes.* Nutrition & Metabolism, 2015. **12**: p. 36.
30. Andersen, S.V., et al., *No effects on appetite or body weight in weight-reduced individuals of foods containing components previously shown to reduce appetite - Results from the SATIN (Satiety Innovation) study.* Obesity Medicine, 2020. **17**.
31. Bomhof, M.R.; Parnell, J.A.; Ramay, H.R.; Crotty, P.; Rioux, K.P.; Probert, C.S.; Jayakumar, S.; Raman, M.; Reimer, R.A. Histological improvement of non-alcoholic steatohepatitis with a prebiotic: a pilot clinical trial. *Eur J Nutr* **2019**, *58*, 1735-1745, doi:https://dx.doi.org/10.1007/s00394-018-1721-2.
32. Bongartz, U., et al., *Flaxseed Mucilage (IQP-LU-104) Reduces Body Weight in Overweight and Moderately Obese Individuals in a 12-week, Three-arm, Double-blind, Randomized and Placebo-controlled Clinical Study.* Obesity Facts, 2022. **07**: p. 07.
33. Calikoglu, F., et al., *The Metabolic Effects of Pre-probiotic Supplementation After Roux-en-Y Gastric Bypass (RYGB) Surgery: a Prospective, Randomized Controlled Study.* Obesity Surgery, 2021. **31**(1): p. 215-223.
34. Cicero, A.F.G., et al., *Psyllium improves dyslipidaemia, hyperglycaemia and hypertension, while guar gum reduces body weight more rapidly in patients affected by metabolic syndrome following an AHA Step 2 diet.* Mediterranean Journal of Nutrition and Metabolism, 2010. **3**(1): p. 47-54.
35. Dewulf, E.M., et al., *Insight into the prebiotic concept: lessons from an exploratory, double blind intervention study with inulin-type fructans in obese women.* Gut, 2013. **62**(8): p. 1112-1121.
36. Genta, S., et al., *Yacon syrup: beneficial effects on obesity and insulin resistance in humans.* Clinical Nutrition, 2009. **28**(2): p. 182-7.
37. Grube, B., et al., *A natural fiber complex reduces body weight in the overweight and obese: a double-blind, randomized, placebo-controlled study.* Obesity, 2013. **21**(1): p. 58-64.
38. Grunberger, G., K.L.C. Jen, and J.D. Artiss, *The benefits of early intervention in obese diabetic patients with FBCx™ - A new dietary fibre.* Diabetes/Metabolism Research and Reviews, 2007. **23**(1): p. 56-62.
39. Guérin-Deremaux, L., et al., *Effects of NUTRIOSE® dietary fiber supplementation on body weight, body composition, energy intake, and hunger in overweight men.* International Journal of Food Sciences and Nutrition, 2011. **62**(6): p. 628-635.

40. Li, S., et al., *NUTRIOSE dietary fiber supplementation improves insulin resistance and determinants of metabolic syndrome in overweight men: a double-blind, randomized, placebo-controlled study.* Applied Physiology, Nutrition, & Metabolism = Physiologie Appliquee, Nutrition et Metabolisme, 2010. **35**(6): p. 773-82.
41. Hassan, O.M.S., et al., *Fiber enrichment of pasta: metabolic effects and diet adherence in obese subjects.* Mediterranean Journal of Nutrition and Metabolism, 2020. **13**(1): p. 53-62.
42. Hess, A.L., et al., *The effect of inulin and resistant maltodextrin on weight loss during energy restriction: a randomised, placebo-controlled, double-blinded intervention.* European Journal of Nutrition, 2020. **59**(6): p. 2507-2524.
43. Benítez-Páez, A., et al., *Sex, Food, and the Gut Microbiota: Disparate Response to Caloric Restriction Diet with Fiber Supplementation in Women and Men.* Molecular nutrition & food research, 2021. **65**(8): p. e2000996.
44. Georg Jensen, M., M. Kristensen, and A. Astrup, *Effect of alginate supplementation on weight loss in obese subjects completing a 12-wk energy-restricted diet: a randomized controlled trial.* American Journal of Clinical Nutrition, 2012. **96**(1): p. 5-13.
45. Kristensen, M., et al., *Supplementation with dairy calcium and/or flaxseed fibers in conjunction with orlistat augments fecal fat excretion without altering ratings of gastrointestinal comfort.* Nutrition & Metabolism, 2017. **14**: p. 13.
46. Pal, S., et al., *Effect on body weight and composition in overweight/obese Australian adults over 12 months consumption of two different types of fibre supplementation in a randomized trial.* Nutrition & Metabolism, 2016. **13**: p. 82.
47. Parnell, J.A. and R.A. Reimer, *Weight loss during oligofructose supplementation is associated with decreased ghrelin and increased peptide YY in overweight and obese adults.* American Journal of Clinical Nutrition, 2009. **89**(6): p. 1751-9.
48. Parnell, J.A., T. Klancic, and R.A. Reimer, *Oligofructose decreases serum lipopolysaccharide and plasminogen activator inhibitor-1 in adults with overweight/obesity.* Obesity, 2017. **25**(3): p. 510-513.
49. Pol, K., et al., *Whole grain and body weight changes in apparently healthy adults: a systematic review and meta-analysis of randomized controlled studies.* American Journal of Clinical Nutrition, 2013. **98**(4): p. 872-84.
50. Reimer, R.A., et al., *Effect of a functional fibre supplement on glycemic control when added to a year-long medically supervised weight management program in adults with type 2 diabetes.* European Journal of Nutrition, 2021. **60**(3): p. 1237-1251.
51. Reimer, R.A., et al., *Inulin-type fructans and whey protein both modulate appetite but only fructans alter gut microbiota in adults with overweight/obesity: A randomized controlled trial.* Molecular Nutrition & Food Research, 2017. **61**(11): p. 11.
52. Reimer, R.A., et al., *Changes in visceral adiposity and serum cholesterol with a novel viscous polysaccharide in Japanese adults with abdominal obesity.* Obesity, 2013. **21**(9): p. E379-E387.
53. Solah, V.A., et al., *Effect of Fibre Supplementation on Body Weight and Composition, Frequency of Eating and Dietary Choice in Overweight Individuals.* Nutrients, 2017. **9**(2): p. 16.
54. Tovar, A.R., et al., *The inclusion of a partial meal replacement with or without inulin to a calorie restricted diet contributes to reach recommended intakes of micronutrients and decrease plasma triglycerides: a randomized clinical trial in obese Mexican women.* Nutrition Journal, 2012. **11**: p. 44.
55. Wood, R.J., et al., *Effects of a carbohydrate-restricted diet on emerging plasma markers for cardiovascular disease.* Nutrition and Metabolism, 2006. **3**.
56. Wood, R.J., et al., *Effects of a carbohydrate-restricted diet with and without supplemental soluble fiber on plasma low-density lipoprotein cholesterol and other clinical markers of cardiovascular risk.* Metabolism: Clinical & Experimental, 2007. **56**(1): p. 58-67.
